# Wearable Standalone Sensing Systems for Smart Agriculture

**DOI:** 10.1002/advs.202414748

**Published:** 2025-03-24

**Authors:** Dongpil Kim, Mohammad Zarei, Siyoung Lee, Hansol Lee, Giwon Lee, Seung Goo Lee

**Affiliations:** ^1^ Department of Horticultural Science Chungnam National University Daejeon 34134 Republic of Korea; ^2^ Department of Chemistry University of Ulsan Ulsan 44610 Republic of Korea; ^3^ Department of Electrical and Systems Engineering University of Pennsylvania Philadelphia PA 19104 USA; ^4^ Department of Chemical and Biological Engineering Gachon University Seongnam 13120 Republic of Korea; ^5^ Department of Chemical Engineering Kwangwoon University Seoul 01897 Republic of Korea

**Keywords:** flexible sensors, noninvasive monitoring, plant monitoring, smart agriculture, wearable sensor

## Abstract

Monitoring crops’ biotic and abiotic responses through sensors is crucial for conserving resources and maintaining crop production. Existing sensors often have technical limitations, measuring only specific parameters with limited reliability and spatial or temporal resolution. Wearable sensing systems are emerging as viable alternatives for plant health monitoring. These systems employ flexible materials attached to the plant body to detect nonchemical (mechanical and optical) and chemical parameters, including transpiration, plant growth, and volatile organic compounds, alongside microclimate factors like surface temperature and humidity. In smart farming, data from real‐time monitoring using these sensors, integrated with Internet of Things technologies, can enhance crop production efficiency by supporting growth environment optimization and pest and disease management. This study examines the core components of wearable standalone systems, such as sensors, circuits, and power sources, and reviews their specific sensing targets and operational principles. It further discusses wearable sensors for plant physiology and metabolite monitoring, affordability, and machine learning techniques for analyzing multimodal sensor data. By summarizing these aspects, this study aims to advance the understanding and development of wearable sensing systems for sustainable agriculture.

## Graphical abstract

This review explores the development and application of wearable sensing systems for plant health monitoring. It examines different types of wearable plant sensors, including their design, working principles, and material choices, with an emphasis on their suitability for real‐world agricultural applications.

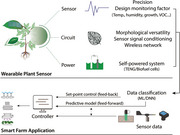

## 1 Introduction

Plants play an important role in providing food, oxygen, and shelter for humans and other living organisms. However, the occurrence of plant diseases due to biological/environmental factors and climate change is accelerating the devastation of agricultural fields, resulting in crop losses and subsequent food shortages in the near future.^[^ ^1^, ^2^ ^]^ Additionally, it is necessary to achieve a 100% increase in global food production by 2050 to fulfill the needs of the rapidly increasing population worldwide.^[^ ^3^, ^4^, ^5^ ^]^ Therefore, the United Nations declared 2020 the “International Year of Plant Health” to emphasize the importance of plant health for human life. To boost crop productivity, numerous plant‐health monitoring technologies have been developed, including robots,^[^ ^6^ ^]^ drones,^[^ ^7^ ^]^ and remote sensing.^[^ ^8^ ^]^ However, these technologies often suffer from limitations such as low spatial and temporal resolution, high operational costs, and limited reliability in complex field environments.^[^ ^9^ ^]^ Wearable sensing systems have emerged as a promising solution for real‐time, continuous monitoring of plant physiological traits, as they can be directly attached to the plant body, enabling more precise measurements.^[^ ^9^ ^]^

Wearable sensing systems were first developed to enhance the quality of human life, with applications such as human healthcare,^[^ ^10^, ^11^ ^]^ electronic skin,^[^ ^12^, ^13^ ^]^ soft robotics,^[^ ^14^, ^15^ ^]^ human‐machine interface,^[^ ^16^, ^17^ ^]^ and artificial prostheses.^[^ ^18^ ^]^ In recent years, wearable sensing systems have been used to monitor plant health by capturing data, such as volatile organic compounds (VOCs) emitted by plants, surface temperature, transpiration, and plant growth, in addition to measuring humidity, environmental temperature, and light. As these factors are closely intertwined to facilitate various plant functions, the analysis of the measured data presents the overall plant‐health status.^[^ ^9^, ^19^, ^20^ ^]^ These systems exhibit outstanding sensing performance, including high sensitivity/selectivity and high mechanical and electrical stability. To practically apply sensors in agricultural fields, wearable sensing systems include sensors integrated with circuits and power sources.^[^ ^21^ ^]^ In other words, acting as standalone sensing systems equipped with independent power generators, these systems detect stimuli via sensors, process/transmit electrical signals with circuits, and use power generators as an energy source. Because there is a lack of conventional energy sources necessary for agriculture worldwide, such sensing systems can be used in conjunction with renewable energy sources such as solar, wind, or rainfall energy.^[^ ^22^, ^23^, ^24^ ^]^ Additionally, the processed electrical signals from the sensing system can be transferred to users over short or long distances via a wireless communication network for numerous purposes.^[^ ^25^ ^]^

With the rapid growth of the Internet of Things (IoT), newly developed industries have been intensively merged with artificial intelligence (AI) to maximize the application of the collected data.^[^ ^26^ ^]^ Machine learning (ML) technology can make predictions based on data obtained from the sensing system and can even provide feedback signals to improve the efficiency of the entire cycle in the system.^[^ ^27^ ^]^ Specifically, in agricultural fields, a standalone sensing system can monitor real‐time plant‐health status and employ ML for providing feedback, thus managing the growth conditions of crops for enhanced crop productivity.^[^ ^9^, ^28^ ^]^ Thus, such processes comprise “smart farming” technology, which facilitates the automatic and precise control of agriculture by using advanced integrated systems. In the near future, state‐of‐the‐art smart farming technologies will have a high potential to resolve the problems of crop loss and food shortage.

Despite the advancements in wearable plant sensors, significant challenges remain unaddressed, particularly in their scalability, durability, energy autonomy, and seamless integration with wireless networks. Existing studies primarily focus on individual sensing components, such as moisture, strain, or pH sensors, but lack a holistic perspective on how these components can be effectively combined into standalone, self‐powered, and remotely accessible systems for large‐scale agricultural deployment. Moreover, the environmental impact of sensor materials, particularly their biodegradability and sustainability, remains a critical yet underexplored area. While some wearable sensors have demonstrated promising results in controlled environments, their long‐term stability, robustness under varying climatic conditions, and adaptability to different crop species require further investigation. Additionally, the potential of AI and ML to enhance sensor performance, optimize data interpretation, and provide predictive insights for precision agriculture is still evolving. Therefore, a comprehensive analysis that bridges these gaps is crucial for advancing the field.^[^ ^29^ ^]^

This review systematically explores the development and application of integrated wearable sensing systems for plant health monitoring. First, it examines different types of wearable plant sensors, including their design, working principles, and material choices, with an emphasis on their suitability for real‐world agricultural applications. Next, it discusses the interface electronics and various energy‐harvesting techniques, such as clean energy, bioenergy, and triboelectric energy harvesting, that enable self‐powered sensor operation. Following this, the review highlights wireless communication technologies, including IoT‐enabled data transmission and networked sensing, which are essential for large‐scale deployment. Additionally, it explores the role of ML in enhancing sensor functionality, improving data analytics, and enabling predictive decision‐making in smart farming. Finally, the review presents current challenges, emerging trends, and future perspectives, offering insights into how wearable plant sensors can be optimized for sustainable, high‐performance agricultural monitoring. By addressing these aspects, this article provides a comprehensive framework for developing next‐generation wearable plant sensors that are efficient, autonomous, and environmentally sustainable.

## 2 Importance of Wearable Standalone Sensing Systems for Smart Farming

**Figure 1:**
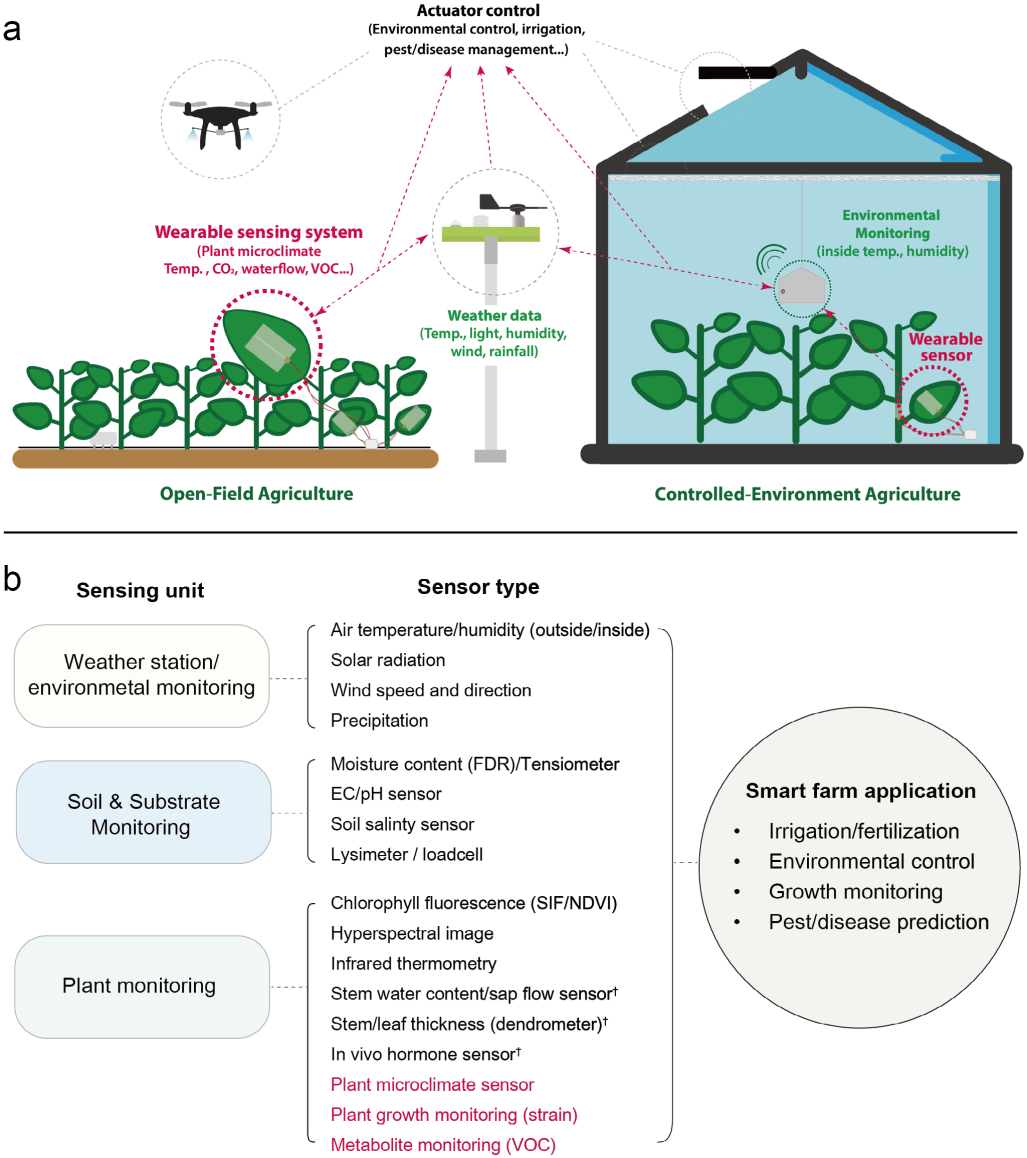
Diagram of the use of wearable sensors in smart farming. a) Schematic of the integration of wearable sensing systems in smart agriculture. b) Sensing units and types currently used in smart farming. “†” indicates sensors similarly that can be developed by wearable sensors, and sensors highlighted in red can be used specifically as wearable sensors. More detailed sensing targets for wearable plant‐monitoring sensors are covered in Section 3.

The fastest and most effective way to improve agricultural productivity in the face of growing global environmental pressures is to increase the efficiency of agricultural resource inputs for producing crops.^[^ ^30^ ^]^ Smart farming broadly refers to the use of IoT technologies to reduce resource use while optimizing crop production quality and quantity. Smart farming has the potential to make agricultural practices more efficient by using technologies such as the IoT, cloud computing, robots, and AI technologies, and by improving the delivery efficiency of fertilizer and plant protection products (PPPs).^[^ ^31^ ^]^ However, chemical fertilizers and PPPs are still overused to increase agricultural production in many regions, and small‐scale farmers, who account for more than 40%, are adding to the environmental burden, particularly due to the lack of monitoring methods and an unsuitable production system.^[^ ^32^, ^33^ ^]^ This can be alleviated by equipping agricultural production systems with real‐time monitoring technology for plants and soil. The challenge in more advanced systems such as greenhouses is to achieve a more efficient circular economy, which involves efficient management of the material flow generated in the entire production process, including management plans for plant byproducts, water, fertilizers, and used nanomaterials.^[^ ^34^ ^]^ In this context, smart farm sensors should be designed to better understand the physiology of crops and their surrounding systems and should be recyclable or degradable after use. IoT includes Internet‐linked devices that share data across networks. In smart farms, this involves the real‐time connection of sensors and control systems such as irrigation systems, robots, drones, greenhouse roofs, curtains, etc. Sensor data are received in real‐time, and various prediction or ML techniques are used to determine specific trends or explain the relationship between the control input and output to make meaningful control decisions.^[^ ^35^ ^]^ Therefore, sensor data play a crucial role in establishing a feedback algorithm for the environmental control of smart farming for optimizing crop production. In the agricultural sector, the primary monitored units are meteorological data from weather stations,^[^ ^36^, ^37^ ^]^ including temperature, humidity, wind direction, wind speed, and rainfall (**Figure** **1**a). In open‐field agriculture, these data provide insight into leaf evapotranspiration and the heat exchange status of the soil,^[^ ^38^, ^39^ ^]^ These data also provide forecast data for irrigation using a reference transpiration model,^[^ ^40^, ^41^ ^]^ In a greenhouse, temperature, humidity, and light levels are controlled by considering internal and external environmental data to reduce energy outflow from the greenhouse and enable the stable and efficient production of crops.^[^ ^42^ ^]^ Therefore, smart farming has been developed with a focus on minimizing biological risks while optimizing energy, water, and nutrient consumption based on primary sensing units.

### 2.1 Current Challenges in Smart Farming

Data collected from systems positioned in areas surrounding the crops are classified as secondary sensing units. These encompass measurements such as temperature, humidity, and irradiance readings from sensors placed near the plants or their surface in the aboveground section. For the root zone area, soil moisture sensors, electrical conductivity (EC) sensors, pH sensors, and lysimeters are used to monitor soil moisture and water and nutrient status of the growing media (Figure 1b). These sensor data represent information regarding the environment surrounding crops and are used to more precisely control the above‐ground environment or nutrient status of the root‐zone. Various methods have been developed to collect appropriate data from open‐field agriculture and controlled‐environment agriculture (CEA) (Figure 1b). For example, soil‐ and substrate‐monitoring sensors have been tailored for integration into the growing system. A cylindrical‐shaped lysimeter is deeply immersed in the soil to monitor evapotranspiration, subsurface water movement, and nutrients in the agricultural ecosystem,^[^ ^43^ ^]^ while a load cell used in CEA environments, such as greenhouses and plant factories, directly measures the crop and substrate weight, EC, and pH of a hydroponic nutrient solution.^[^ ^44^, ^45^ ^]^ In different smart‐farm environments, each sensor is designed to collect the appropriate data to implement an irrigation system.

Although secondary sensors reduce biological risk by monitoring the plant's surrounding environment, this does not necessarily indicate the optimal delivery of resources to the crop. Owing to gaps in the understanding of plant physiology and conventional crop management, there are challenges in implementing precisely controlled agriculture. In traditional agriculture, optimal growth conditions, such as temperature, CO_2_, and water status, have been derived from empirical experiments, such as destructive or nondestructive investigations of crop growth (e.g., yield, dry mass) or measurement techniques (e.g., gas exchange measurement and chlorophyll content).^[^ ^46^, ^47^, ^48^, ^49^ ^]^ However, these results cannot be used to ensure optimal growth conditions throughout the cropping season because they do not consider the real‐time physiological response of the crop.

In other words, the bottleneck in optimizing smart‐farm cultivation is the feedback of crop health information to sensors. Consequently, sensors capable of closely assessing plant conditions have been integrated into smart farming, and these sensors are referred to as plant‐based or plant‐monitoring sensors (Figure 1b).^[^ ^50^, ^51^ ^]^ Plant‐monitoring sensors include sap flow sensors, dendrometers, infrared cameras, hyperspectral imaging, etc. These sensors determine the status of plants by being physically mounted on the plant surface or by conducting measurements optically. They can nondestructively monitor plant responses over time or observe a wide area using camera systems. Using image sensors such as leaf temperature sensors (thermal imaging) or hyperspectral cameras is advantageous for evaluating plant status in a wide‐ranging, nondestructive manner.^[^ ^52^, ^53^ ^]^ Plant‐monitoring sensors provide background knowledge for environmental control by reflecting information regarding plants, however, there remains a considerable need for improving the existing sensors to ensure the optimal delivery of resources to crops and disease prevention.

### 2.2 Necessity of Wearable Sensors for Smart Farming

The first advantage of wearable electronics is the collection of plant‐health data without restrictions on the attachment site. This is because of the use of flexible materials for constructing wearable sensors, thus enabling them to establish conformal contact with the plant surface. When wearable sensors were first introduced for plant monitoring, they were employed to collect plant‐health data in a similar fashion to previously used sensors (Figure 1b, dagger footnote).^[^ ^54^, ^55^ ^]^ For example, a dendrometer‐like sensor was developed by measuring the strain in fruit expansion,^[^ ^56^ ^]^ and sap flow data were collected by attaching a wearable sensor to the stem to analyze water transport.^[^ ^57^ ^]^ Because wearable sensors are designed using flexible polymers and composites, they provide plant‐health data conveniently through adaptable and noninvasive methods. In contrast, the existing probe‐type sensors can only be applied to hard stems for robust data collection. Furthermore, regarding data continuity, wearable sensors collect data in a nondestructive manner throughout all stages of plant growth, from the juvenile to adult stages. This indicates the potential scalability of wearable sensors for plant monitoring without time and space constraints. The second advantage is the multifunctionality of the wearable sensors. Wearable sensors can replace traditional plant‐monitoring sensors and enable new ways of measuring data (strain, microclimate, and VOCs) efficiently (Figure 1b). Plant growth and microclimate data are key information for the efficient operation of smart farms, consequently, smart farms utilize the correlation between climate data obtained from currently operational sensors and actual leaf microclimate and plant growth data (Figure 1a). Multimodal sensors can simultaneously collect leaf microclimate data such as temperature, humidity, and light, thus facilitating the measurement of leaf adaxial microclimate changes in real‐time.^[^ ^9^ ^]^ Simultaneously, wearable plant growth sensors continuously monitor cell elongation by attaching strain sensors to the stems, leaves, and fruits.^[^ ^25^, ^58^, ^59^ ^]^ They can collect phenotypic data stably, regardless of the sensor attachment location, weather changes, and external stimuli, because of their versatility. Crop growth management decisions aim to balance plant growth and photosynthate partitioning (source‐sink balance), which is determined by the number of yield components (fruit number, pruning).^[^ ^60^, ^61^ ^]^ Traditionally, plant growth measurement has been conducted manually or with RGB images, this can be labor‐intensive and has data resolution, accuracy, and discontinuity issues.^[^ ^62^ ^]^ Wearable strain sensors can individually measure plant organ development to determine source‐sink demands at different stages of fruit development, further, they can be used in various ways to manage light and temperature for balanced cultivation.^[^ ^63^ ^]^ Therefore, microclimate and strain sensors are the first commercialized wearable devices for smart farms to help growers identify optimal growing conditions and detect stress responses early.

Wearable sensors can integrate physical and chemical sensing modules to improve our understanding of crop physiology in plant production systems. Water transport in plants has been extensively studied in ecology and plant physiology for decades.^[^ ^64^ ^]^ The process of transporting water and nutrients to the under‐ and aboveground segments of plants while distributing assimilated products to each organ is an intricate process that is affected by various factors. Stomata, the final pathways of water movement, are the first responders that sense and react according to environmental changes to regulate water intake.^[^ ^65^ ^]^ Plant responses to abiotic factors, such as drought, CO_2_, temperature, and light, occur on different spatiotemporal scales, starting from short‐term stomatal activation via plant hormones, such as abscisic acid (ABA) to finally extend to water potential changes in stems and roots.^[^ ^66^ ^]^ Studies up to the early 2000s modeled and analyzed the biophysical reactions of plants on an individual organ scale.^[^ ^67^, ^68^, ^69^ ^]^ However, a model that can comprehensively analyze plants and their surroundings has become necessary because of the environmental changes due to climate change, such as drought and heat waves. In the context of integrated water movement, Carminati and Javaux investigated hydraulic limitations arising from soil water availability.^[^ ^70^ ^]^ Concurrently, a plant hydraulic model was introduced to elucidate the relationship between above and underground plant components and their correlation with stomatal responses and photosynthesis.^[^ ^71^ ^]^ By affixing wearable sensors to plant surfaces, researchers can directly analyze how factors, such as stomatal occlusion and xylem embolism, limit water transport. One characteristic application of wearable sensors is the detection of plant‐emitted chemical signals for monitoring plant health.^[^ ^72^ ^]^ Such chemical signals (e.g., H_2_O_2_, Ca^2+^, nitric oxide, and ABA) from external stresses can be seamlessly converted into digital data by integrating sensing particles into wearable sensors.^[^ ^73^ ^]^ Incorporating these features facilitates a multifunctional wearable sensor to continuously analyze the relationship between vapor pressure deficit, transpiration, and salicylic acid in the leaf epidermis.^[^ ^74^ ^]^ This innovative approach deepens our understanding of the physiological processes of plants and paves the way for the development of new models across diverse research fields for various purposes.

We can broadly divide the development of wearable sensors into two directions: 1) sensors for understanding plant physiology and growth processes, and 2) sensors for large‐scale deployment in actual farming. Wearable sensors are ideal for smart farming because they are self‐sustained robust systems comprising multiple sensing modules, substrates, and power sources mounted on flexible materials.^[^ ^75^ ^]^ Currently, research on wearable plant sensors is limited as it has been fragmented and, in most cases, has stopped at monitoring the physiological responses or growth processes of plants. This indicates that several barriers must be overcome before wearable sensors can support actual smart farming systems and enable large‐scale deployments. These barriers include sensor durability, wireless communication, multi‐modal functionality, and the comprehensive interpretation of data collected from multiple sensors. Because smart‐farm sensors are often subjected to physical stimuli such as harsh weather or unexpected stimuli and impacts from agricultural machines, harvesting, pesticide spraying, wild animals, etc., they must be small, lightweight, and capable of reliable measurements with physically robust design for long‐term use. A smart farm should facilitate the connection of multiple sensors for controlling actuators, emphasizing real‐time monitoring via wireless communication.^[^ ^76^ ^]^Wearable standalone sensing systems have implemented elemental technologies for smart agriculture sensors by including features such as multiple plant‐sensing modules, circuits, wireless communication, and self‐powered in a small size.^[^ ^77^ ^]^ The development of these technologies will accelerate the commercialization of wearable sensors for large‐scale use on smart farms. In addition, the ‘transient electronics’ technology is an eco‐friendly electronic device that naturally degrades at the cultivation site after use, which may present another possibility for wearable sensors to realize fully circular agriculture.^[^ ^78^, ^79^ ^]^

## 3 Plant‐Monitoring Wearable Electronics

The growing interest in wearable plant sensors reflects the potential for precision agriculture and sustainable farming practices. While wearable sensors have demonstrated promise in both research and field applications, their design and deployment differ depending on their intended function. This distinction is important for advancing agricultural technology and ensuring that these sensors meet the needs of farmers and researchers alike. In this context, it is essential to understand the two main functions of wearable plant sensors including sensors for understanding plant physiology and growth processes, and sensors for large‐scale deployment in the fields.

1. Sensors for understanding plant physiology and growth processes: These sensors are primarily used for research purposes to monitor plant health, detect environmental stressors, and better understand the physiological processes involved in growth. These sensors often focus on high precision and sensitivity to capture detailed biological signals, such as leaf wetness, stomatal conductance, or nutrient uptake. For instance, sensors integrated with wireless systems have been used for monitoring plant growth conditions in controlled environments, offering insights into early plant stress detection and physiological responses.^[^ ^80^, ^81^ ^]^ The technologies used here often require sophisticated sensors that can measure specific parameters like moisture content, temperature, and leaf behavior at high resolutions, which are crucial for understanding plant physiology at a deeper level.^[^ ^9^, ^82^ ^]^
2. Sensors for large‐scale deployment in the field: In contrast, sensors used for large‐scale agricultural applications must be durable, cost‐effective, and capable of withstanding harsh field conditions.^[^ ^83^, ^84^ ^]^ These sensors are designed to be deployed across extensive agricultural land and are often integrated into IoT‐based systems to monitor environmental parameters such as soil moisture, temperature, and nutrient levels on a large scale.^[^ ^83^ ^]^ These sensors are typically less complex than those used for physiological research, focusing instead on affordability and robustness. Low‐cost sensors, such as capacitive or resistive moisture sensors, are widely used for agricultural applications to monitor soil moisture in real‐time, contributing to water‐use efficiency and precision irrigation.^[^ ^80^, ^81^ ^]^ Additionally, biodegradable sensors are becoming an increasingly viable solution for field‐deployment, offering sustainability by decomposing after their intended use.^[^ ^81^, ^85^ ^]^ The technologies for these two applications can indeed differ in terms of complexity, cost, and durability. For plant physiology monitoring, sensors may require high accuracy and integration with other complimentary sophisticated platforms. In contrast, sensors designed for field deployment emphasize scalability, cost‐effectiveness, and the ability to function under varied environmental conditions.

### 3.1 Wearable Sensors for Plant Monitoring

The quality and growth of plants are significantly affected by humidity and temperature levels.^[^ ^86^, ^87^, ^88^, ^89^ ^]^ Hence, while maintaining ideal environmental conditions for each crop and plant within the greenhouse is beneficial, particularly with respect to humidity, this is a complex task. The microclimate surrounding each plant is influenced by its position and response to local environmental conditions. For example, as temperature changes, humidity levels fluctuate, and plants continually release moisture into their surroundings through transpiration, this transpiration rate depends on the local temperature. Temperature and humidity levels also profoundly impact the process of photosynthesis, wherein carbon dioxide and water are converted into sugars for energy and subsequently used for plant growth. Hence, plant growth relies heavily on conditions that support optimal photosynthesis, which is in turn profoundly influenced by the delicate balance between humidity and temperature. Therefore, to ensure the well‐being of plants, it is essential to continually monitor the factors that influence and are influenced by their growth. This section explores the development of wearable sensors designed for plant monitoring and the challenges associated with their development. Wearable plant sensors can potentially replace traditional methods such as remote or image‐based monitoring approaches.^[^ ^86^ ^]^ In this section, we will discuss the application of various types of plant sensors, such as those used for monitoring plant growth and the surrounding microenvironment, and for measuring metabolites. Additionally, examples of each application will be discussed in detail.

#### 3.1.1 Wearable Sensors for Microclimate Monitoring

**Figure 2:**
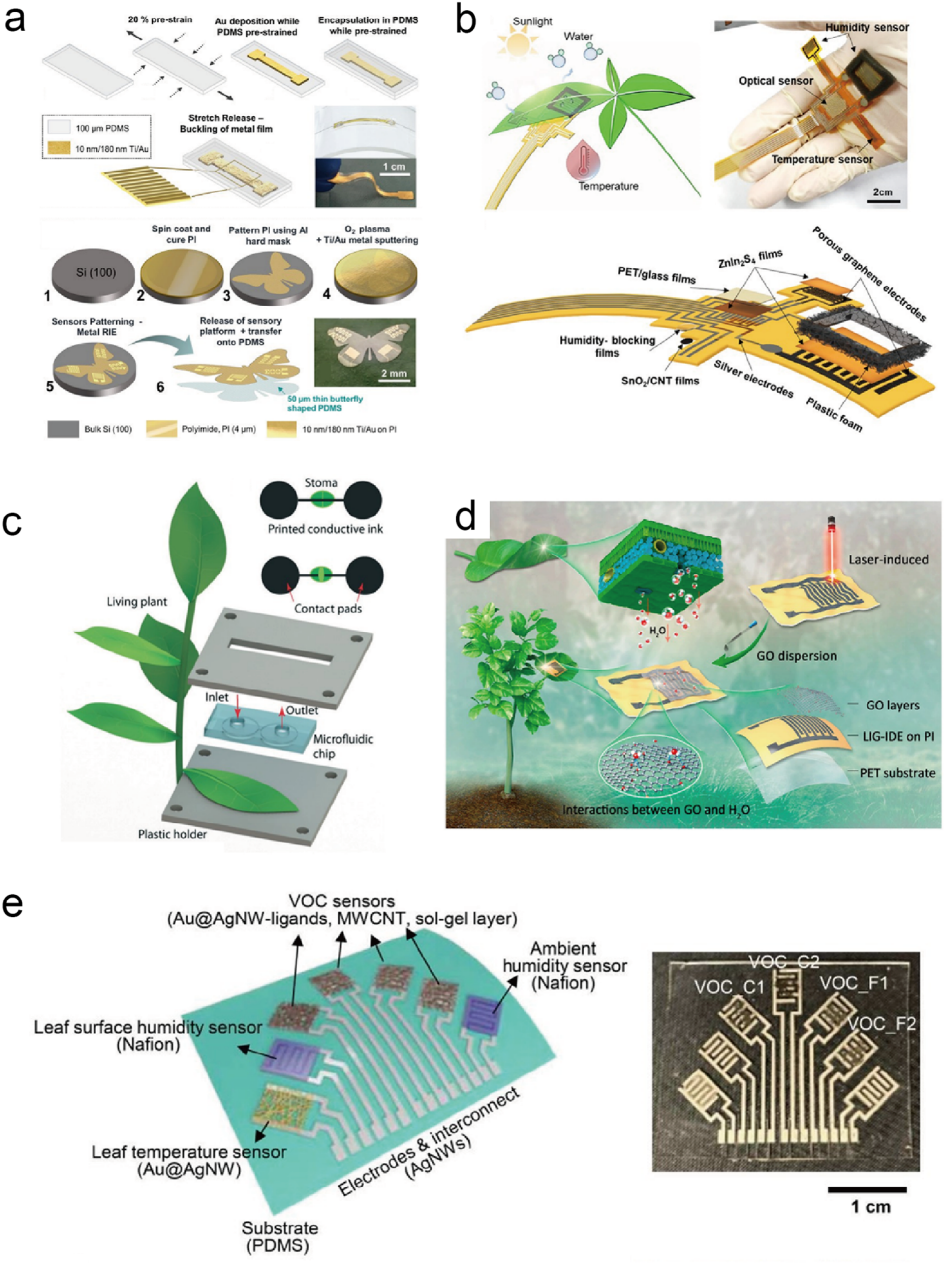
Design and structure of plant sensors measuring microclimate factors. a) Stretchable butterfly strain sensor. Reproduced with Permission.^[^ ^21^ ^]^ Copyright 2018, Nature Publishing Group. b) Multimodal flexible plant sensor. Reproduced with Permission.^[^ ^20^ ^]^ Copyright 2020, American Chemical Society. c) Schematic of the printed microsensor featuring two contact pads and a stripe that extends over a single stoma. Reproduced with Permission.^[^ ^93^ ^]^ Copyright 2017, Royal Society of Chemistry. d) Materials and fabrication strategy of the flexible humidity sensor. Reproduced with Permission.^[^ ^94^ ^]^ Copyright 2020, Elsevier BV. e) Multimodal wearable plant sensor for temperature, humidity, and VOC sensing. Reproduced with Permission.^[^ ^9^ ^]^ Copyright 2023, American Association for the Advancement of Science.

Traditional climate monitoring relies predominantly on centralized sensors that measure temperature and humidity. However, there is a growing emphasis on embracing smart or precision agricultural approaches to sustainably enhance crop yields by remotely monitoring the microenvironment of plants and employing integrated decision‐making processes to optimize the efficient allocation of scarce resources. In 2018, Nassar et al. developed a flexible multisensory platform that integrated temperature and humidity sensors on the same flexible and ultralight butterfly‐shaped polyimide (PI)/polydimethylsiloxane (PDMS) platform for plant microclimate monitoring (**Figure** **2**a).^[^ ^21^ ^]^ The developed sensors exhibited linear responses and demonstrated satisfactory sensitivity for monitoring the surrounding microclimate changes. The reported temperature sensitivity was in line with previous studies, falling within the reported range of 0.0018–0.0061 °C.^[^ ^90^ ^]^ The humidity sensor exhibited high sensitivity when compared to polyimide‐based humidity sensors, with sensitivities as low as 0.0038% relative humidity (RH)^[^ ^91^ ^]^ and 0.0025% RH.^[^ ^92^ ^]^ The RH sensing mechanism relies on the humidity‐dependent capacitance of the polyimide, whereas temperature sensing is based on a meander‐structured gold (Au) thermistor. Additionally, the developed sensing platform was linked to a rechargeable battery and chip for storing and wirelessly transmitting sensed data to a smartphone. Based on the abovementioned configuration, the developed platform can be used for the real‐time monitoring of temperature and RH. Lu et al. developed an integrated multimodal flexible sensor system for managing plant growth (Figure 2b) based on stacked ZnIn_2_S_4_ (ZIS) nanosheets as the core sensing medium.^[^ ^20^ ^]^ This ZIS‐based flexible sensor provided rapid response capabilities, detecting light illumination within ≈4 ms and maintaining a consistent and long‐lasting performance in humidity monitoring, a feature previously not included in other comparable sensor technologies. Additionally, the developed system could visually record instances of dehydration over an extended monitoring period exceeding 15 days. The use of ZIS nanosheets as sensor layers ensured stable and enduring humidity sensing, covering a wide moisture range of up to 90%.

#### 3.1.2 Wearable Sensors for Plant Physiology Monitoring

Enhancing the spatiotemporal resolution of plant physiological monitoring is essential for gaining a more comprehensive understanding of the underlying biological mechanisms. Traditional methods generally use destructive techniques that involve the collection of plant tissues, subsequent processing, and analysis through chromatography/mass spectrometry. However, various types of point‐of‐care and wearable sensors have been introduced in recent years,^[^ ^86^ ^]^ including implantable devices and epidermal/wearable sensors that can be attached to leaves or stems, ensuring no/minimal destruction. The monitoring of stomatal conductance, which assesses the extent of stomatal openings, determines a plant's water consumption and provides insights into its water status. For instance, under drought conditions, stomata minimize transpiration and prevent water loss, however, this simultaneously reduces the photosynthetic rate and inhibits plant growth. By measuring transpiration and carbon assimilation, we can calculate a plant's water use efficiency, which is a valuable metric for evaluating a plant's productivity while conserving water resources. State‐of‐the‐art transpiration analyzers, represented by handheld gas exchange devices, can be employed even under field conditions. Attached to a designated leaf area, these instruments measure water vapor, temperature, and carbon dioxide, enabling the calculation of transpiration rate and stomatal conductance. Additionally, these gas exchange devices can be augmented with a fluorometer to quantify chlorophyll fluorescence, a useful indicator of plant health. However, reliable data collection requires thorough calibration of the equipment, and the fluorometer chamber's conditions must mirror the ambient environment. Furthermore, this instrument tends to be costly and cumbersome and poses challenges when analyzing multiple plants simultaneously. These constraints highlight the need for the development of methods and approaches for real‐time, long‐term monitoring of stomatal function in a plant's natural growth environment. Koman et al.^[^ ^93^ ^]^ employed a nanoparticle‐based conducting ink to construct a highly durable electrical conductometric sensor that is activated by the stomatal pore itself. This activation was repeated and reversed for a period of over 1 week (Figure 2c). This specialized sensor enables real‐time monitoring of single stomatal opening and closing times within plants. The authors observed that opening times ranged from 7.0 ± 0.5 min to 25.0 ± 0.5 min and closing times ranged from 53.0 ± 0.5 min to 45.0 ± 0.5 min in *Spathiphyllum wallisii*. Notably, a single stoma in *Spathiphyllum wallisii* was capable of distinguishing incident light intensities (up to 12 mW cm^−2^) with a rapid temporal latency of as little as 7.0 ± 0.5 min. Throughout the 7‐day period, the latency in opening and closing times remained consistent over the plant's daily cycle and gradually increased with the onset of drought. Lan et al.^[^ ^94^ ^]^ proposed a practical, efficient, and robust approach for the mass production of flexible and wearable humidity sensors (Figure 2d). They leveraged laser direct writing technology to create laser‐induced graphene interdigital electrodes (LIG‐IDE) on PI film. By employing graphene oxide as a humidity‐sensitive material, a flexible capacitive‐type humidity sensor was constructed, which exhibited low hysteresis, high humidity sensitivity (3215.25 pF/% RH), and long‐term stability (with a variation of less than ±1%). This sensor can be directly affixed to plant leaves (*Epipremnum aurem*) for real‐time, extended monitoring of transpiration from the stomata without causing harm to the plant. In 2023, Lee et al. developed a wearable multimodal leaf surface‐mounted sensor for monitoring a plant's biochemical and biophysical signals and their microenvironment (Figure 2e).^[^ ^9^ ^]^ Their sensing system included a unified platform that integrated sensors for detecting VOCs, temperature, and humidity. The selection of the abaxial leaf surface as the attachment position was based on stomatal density, thus enhancing the signal strength of the sensors. This versatile system facilitated various stress‐monitoring applications, spanning from monitoring plant water loss to the early identification of plant pathogens.

**Figure 3:**
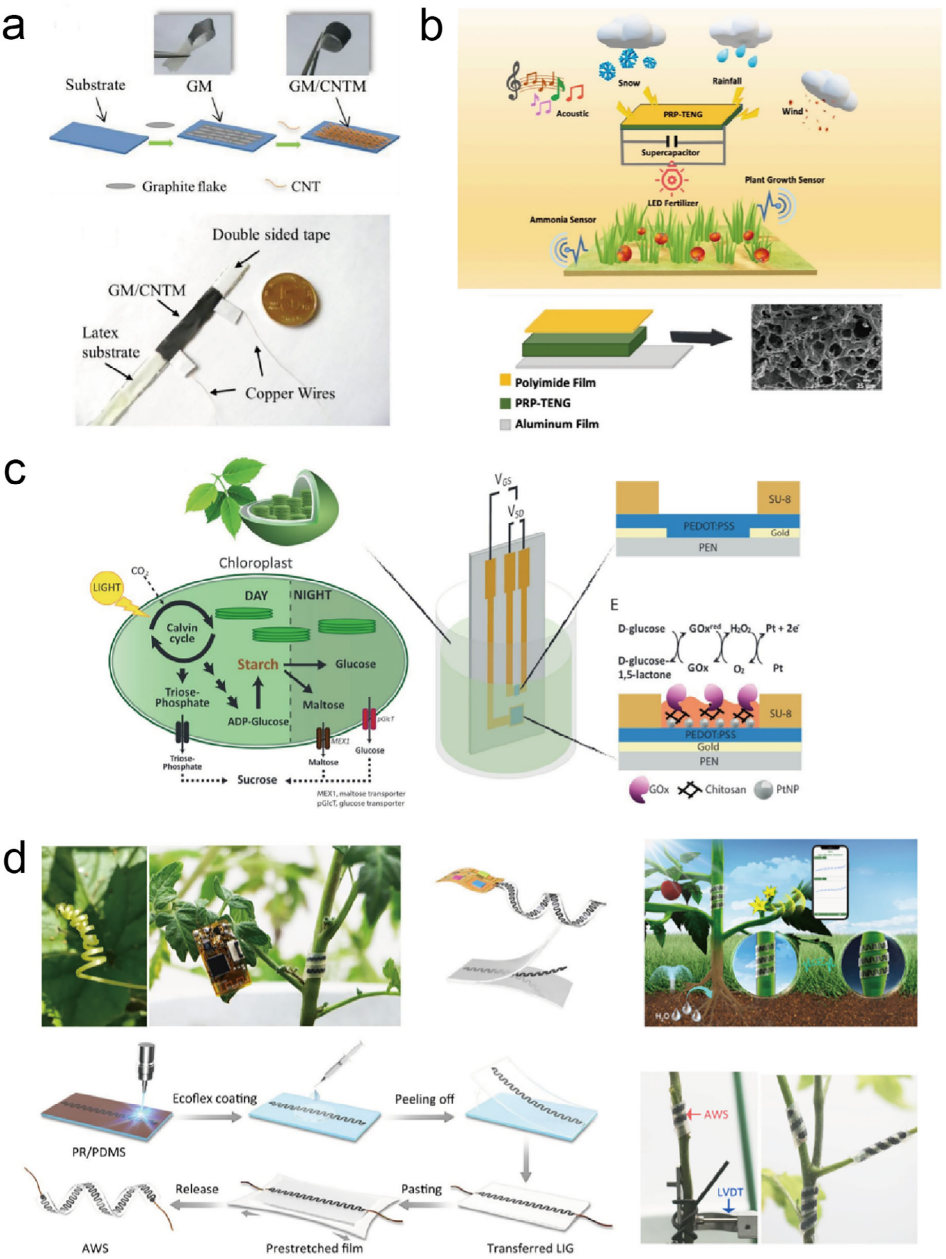
Structure and design of physiological and metabolite sensors. a) Structure of graphite membrane/CNT membrane plant growth sensor (GM/CNTM). Reproduced with Permission.^[^ ^59^ ^]^ Copyright 2019, Elsevier BV. b) Schematic of the PAA‐RGO‐PANI plant growth sensor. Reproduced with Permission.^[^ ^95^ ^]^ Copyright 2021, Elsevier BV. c) Schematic of the organic electrochemical transistor glucose sensor applied to the plant. Reproduced with Permission.^[^ ^96^ ^]^ Copyright 2020, Wiley. d) Fabrication and structure of the plant stem's pulse (expansion and shrink) monitoring sensor. Reproduced with Permission.^[^ ^25^ ^]^ Copyright 2022, Nature Publishing Group.

Plant growth can be electrically monitored using wearable and stretchable resistive sensors that exhibit alterations in resistivity as they undergo strain. These sensors can be affixed to actively growing regions of the plant, ensuring that as the plant tissue elongates, the resistance of the sensor undergoes corresponding modifications. Tang et al.^[^ ^59^ ^]^ developed a simple fabrication process for the development of wearable strain sensors for highly sensitive, automated, and real‐time monitoring of plant growth ranging from nanometers to centimeters (**Figure** **3**a). Using the deposition of graphite ink and carbon nanotube (CNT) ink, their subsequent solidification under ambient conditions, and harnessing the combined reinforcing properties of graphite and CNT membranes, a flexible, stretchable, and wearable CNT/graphite sensor was developed. The obtained sensor was integrated into an all‐in‐one device that included a custom‐made readout circuit for real‐time measurement of plant growth. This device was used to successfully perform real‐time monitoring of *Solanum melongena L*. and *Cucurbita pepo* by monitoring the distinct rhythmic growth patterns in their fruits. Hsu et al. developed a piezoelectric‐resistive pyroelectric triboelectric nanogenerator (PRP‐TENG) capable of harnessing various clean energy sources, including acoustic, rainfall, and wind energy, and efficiently converting them into electrical power based on a hydrogel consisting of a double network comprising polyacrylic acid (PAA), reduced graphene oxide (RGO), and polyaniline (PANI), for a range of applications such as green agriculture and smart farming systems (Figure 3b).^[^ ^95^ ^]^ This PRP‐TENG achieved a power density of 424 mW m^−2^, an open‐circuit voltage of up to 21.77 V, and a current output of up to 48.69 µA. In this device, a PAA‐RGO‐PANI supercapacitor, acting as an energy storage device, enables a continuous power supply for LED lights designed to promote plant growth. Remarkably, the capacitance of the PAA‐RGO‐PANI supercapacitor remained stable at 2330 mF cm^−2^ within 5000 cycles, ensuring prolonged functionality. The PAA‐RGO‐PANI hydrogel exhibited exceptional mechanical properties with a stress tolerance of 1050 KPa and an impressive strain capacity of 650%. These sensors maintain a high degree of sensitivity for monitoring plant growth for extended periods of up to 18 days.

#### 3.1.3 Wearable Sensors for Metabolite Monitoring

In the analyses of plant metabolite monitoring data, biochemical analysis is typically conducted using methods such as capillary electrophoresis mass spectrometry (CE‐MS), Fourier transform ion cyclotron resonance mass spectrometry (FTICR‐MS), liquid chromatography‐mass spectroscopy (LC‐MS), gas chromatography‐mass spectrometry (GC‐MS), ion mobility spectrometry, matrix‐assisted laser desorption/ionization, and nuclear magnetic resonance (NMR).^[^ ^97^ ^]^ Although these approaches are dependable and can achieve extremely low detection limits, they lack the capacity for in vivo and real‐time measurements. Despite the subcellular precision offered by these techniques, their detection depends on the use of bulky instruments, thereby limiting their application on farms.^[^ ^96^, ^98^ ^]^ In one study, Diacci et al.^[^ ^96^ ^]^ developed an organic electrochemical transistor (OECT) glucose sensor that was applied to chloroplasts (wild‐type tobacco plants) for the real‐time monitoring of glucose export from chloroplasts during two distinct metabolic phases, offering precise quantification of transfer dynamics with a remarkable time resolution of 1 min (Figure 3c). The OECT gate was biofunctionalized with glucose oxidase (GOx) cross‐linked within a chitosan matrix. The results showed that glucose was only detected in chloroplasts harvested during the night, aligning with the existing knowledge of the starch degradation process. Additionally, Diacci et al.^[^ ^98^ ^]^ developed an implantable OECT enzymatic biosensor for real‐time monitoring of sugar fluctuations within the vascular tissue of trees. The authors successfully observed previously uncharacterized diurnal changes in sucrose levels in the xylem sap of the greenhouse‐grown hybrid aspen (*Populus tremula × tremuloides*). Zhang et al.^[^ ^25^ ^]^ inspired by the natural adaptability of plant tendrils, developed an integrated plant wearable system (IPWS) for monitoring plant pulses through an adaptive winding strain (AWS) sensor (Figure 3d). The AWS sensor conformably encircled the tomato stem and was selected for investigation. Additionally, the AWS sensor exhibited a high resistance to temperature fluctuations. The results showed that IPWS could be used for wireless monitoring of the expansion and shrinkage of plant stems, which are indicators of the growth and water status of tomato plants, further showing that IPWS could be used to monitor other plants.

#### 3.1.4 Cost, Affordability, and Biodegradability of Plant Monitoring Sensors

Wearable plant monitoring platforms differ from human health monitoring sensors, which have long been developed using existing technologies for strain, pH, and moisture sensing. The challenge in agricultural sensing platforms is that these sensors are often expensive and may contain environmentally hazardous materials. Since retrieving field‐deployed sensors is often impractical, these sensors must be biodegradable, efficient, and low‐cost. The affordability of sensors depends on factors such as measurement technique, accuracy, connectivity, durability, and fabrication costs.^[^ ^99^ ^]^ More accurate sensors tend to be expensive due to high‐quality components, sophisticated calibration, and advanced fabrication methods. Sensors with wireless connectivity or rugged designs also cost more.^[^ ^100^ ^]^ Commercial soil moisture sensors can be expensive, labor‐intensive, and limit deployment density. Capacitive sensors are considered low‐cost with high precision and meet the requirements of wireless sensor networks.^[^ ^101^, ^102^ ^]^ Various low‐cost sensors have been developed using techniques like time‐domain reflectometry, heat dissipation, electrical impedance spectroscopy, and radio frequency technology.^[^ ^103^, ^104^, ^105^ ^]^ Smart sensing systems, including IoT‐based solutions, enhance water‐use efficiency in precision agriculture but may contribute to environmental pollution.^[^ ^106^ ^]^ To minimize their ecological impact, biodegradable sensors offer a sustainable alternative, functioning throughout cropping seasons before decomposing in the soil. To reduce environmental impact, non‐biodegradable substrates can be replaced with sustainable alternatives like poly(lactic acid) (PLA) fiber,^[^ ^107^ ^]^ bacterial cellulose,^[^ ^108^ ^]^ poly(3‐hydroxybutyrate‐co‐3‐hydroxyvalerate) (PHBV),^[^ ^109^ ^]^ beeswax^[^ ^106^, ^110^ ^]^ or cellulose acetate (CA).^[^ ^102^ ^]^ These eco‐friendly materials offer advantages such as low cost, flexibility, biocompatibility, and biodegradability, making them suitable for wearable sensors and biosensors.

In one study, Kurth et al.^[^ ^80^ ^]^ developed a monitoring system for early detection of adverse plant growth conditions. The hardware includes a wireless sensor network for data collection and transfer, a gateway for internet connectivity, and a central server running an expert system. These components were fabricated using biodegradable or inert materials with minimal metal and ceramic content, allowing them to remain in the field after harvesting. Single‐chip controllers with built‐in radio communication are integrated into printed circuit boards fabricated partly from conventional printed circuit boards (PCBs) materials and biodegradable substrates. Wiring and communication antennas are printed onto these substrates, and a biodegradable zinc‐manganese dioxide battery powers the sensor nodes. The key advantage of this system is its affordability and degradability, enabling high‐density microclimate monitoring at a lower cost and effort. This facilitates site‐specific treatments, reducing the use of plant protection agents at the field scale. The sensor nodes use single‐chip controllers with built‐in radio interfaces on printed circuit boards made partly of conventional PCB material and cellulose substrates. Several components are made from environmentally friendly materials with minimal soil impact, including the primary battery, antenna, and feed line, electrical wiring, soil moisture sensor, and housing. Power is supplied by a printed zinc‐manganese dioxide primary battery (4.5V, 80 mAh). Since antenna and wiring printing utilize roll‐to‐roll processes, costs are significantly lower than traditional PCB manufacturing. In another study, Rahimi et al.^[^ ^81^ ^]^ developed a fully degradable Intelligent Radio Transmitting Sensor (DIRTS) for remote subsoil moisture monitoring using drone‐assisted wireless sensing. The device features a miniaturized resonating antenna encapsulated in a biodegradable polymer, with its resonant frequency varying based on the soil's dielectric properties. DIRTS' simple design enables scalable, cost‐effective additive manufacturing, allowing for automated soil distribution. A biodegradation study confirms stability for up to one year, with less than 4% sensitivity change before degradation begins. The authors claimed DIRTS represents a step toward sustainable precision agriculture with minimal environmental impact. The integration of eco‐friendly biopolymeric films with printed devices enables the development of plant‐wearable sensors for decentralized pesticide analysis in precision agriculture and food safety.

### 3.2 Interface Circuits

**Figure 4:**
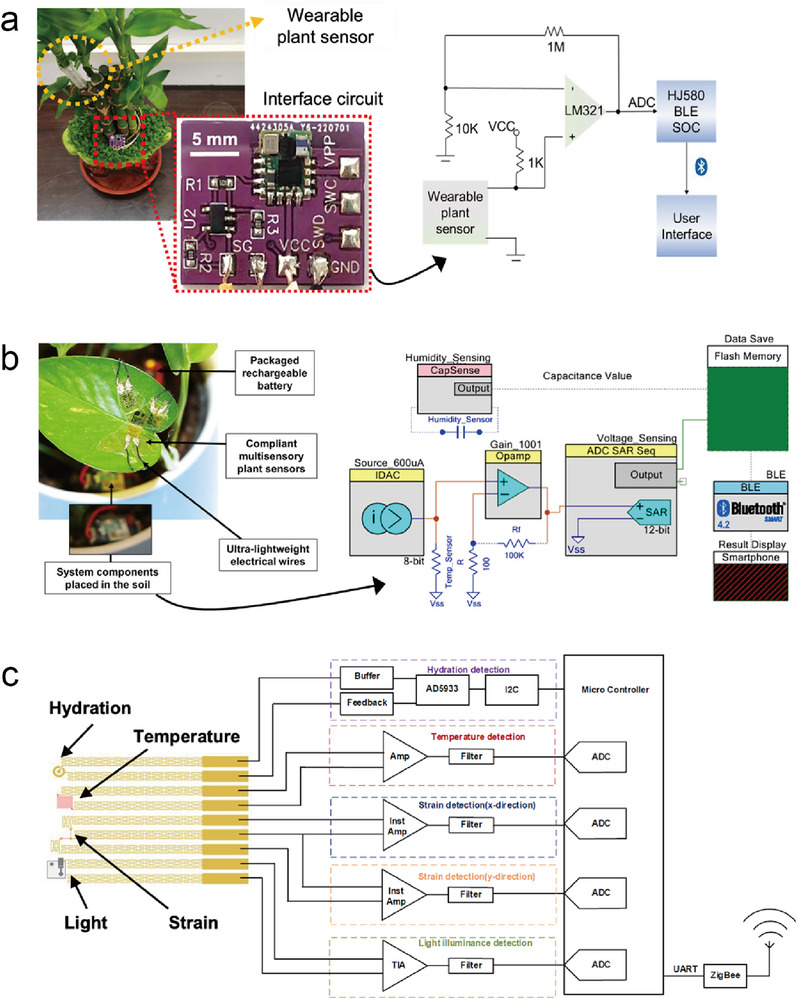
Interface circuit diagrams of wearable standalone plant‐sensing systems, including wireless networks. a) A wearable plant growth sensor with the interface circuit lying on the pot ground (left). Corresponding circuit diagram (right). Reproduced with Permission.^[^ ^58^ ^]^ Copyright 2023, American Chemical Society. b) Circuit diagram of a plant wearable device for monitoring microclimate factors. Reproduced with Permission.^[^ ^21^ ^]^ Copyright 2018, Nature Publishing Group. c) Circuit diagram of a multimodal plant sensor. Reproduced with Permission.^[^ ^116^ ^]^ Copyright 2019, American Chemical Society.

A standalone sensing system requires a real‐time electrical signal monitoring interface circuit that is capable of wireless data transmission, data visualization, and storage. In the pursuit of wearable systems, it is advantageous not only for the sensor but also for the interface circuit to exhibit flexibility, stretchability, surface attachability, miniaturization, or biodegradability. However, achieving full stretchability, flexibility, and biodegradability in all circuit components remains technically challenging.^[^ ^111^, ^112^ ^]^ While microfabrication technology enables integrated circuit production in chip form, the commercial viability of wearable sensing systems should first be validated. Consequently, most researchers have concentrated on proving the functional feasibility of interface circuits by integrating circuit components, which can be readily purchasable for several dollars,^[^ ^57^ ^]^ on rigid printed circuit boards (PCBs). These interface circuits were often situated at a physically long distance from the wearable sensor (**Figure** **4**a,b, left). To emphasize circuit wearability, researchers have employed flexible PCBs^[^ ^25^, ^57^ ^]^ or streamlined interface circuits to operate via remote power reception.^[^ ^113^, ^114^, ^115^ ^]^

#### 3.2.1 Amplifier and Analog‐To‐Digital Converter

Typically, wearable electronic sensors yield analog output signals that provide a direct representation of input parameters. Nevertheless, these analog signals are susceptible to distortion owing to noise, interference, and degradation over long transmission distances. In some instances, their small amplitude makes it challenging to differentiate them from the background or electrical noise, and they are incompatible with modern digital electronics. Consequently, the interface circuits of most wearable standalone plant sensors employ operational amplifiers (op‐amps) and analog‐to‐digital converters (ADCs) for signal amplification, noise filtering, and conversion to a digital format (Figure 4). Op‐amps amplify weak analog sensor signals. Most of them feature two input terminals (inverting and noninverting) and a single output terminal, as illustrated in “LM321” in Figure 4a, right,^[^ ^58^ ^]^ “Op‐amp” in Figure 4b, right,^[^ ^21^ ^]^ and “Amp” in Figure 4c.^[^ ^116^ ^]^ Typically, their power supply terminals (+ and −) are omitted from circuit diagrams. Op‐amps amplify the voltage difference between the two input terminals, known as the gain.^[^ ^117^, ^118^ ^]^ In wearable plant‐sensing systems, when the sensor presents a variable impedance (e.g., capacitance^[^ ^119^ ^]^ or resistance^[^ ^58^ ^]^), it is connected to one input alongside the voltage/current source. Alternatively, when the sensor generates a voltage (e.g., piezo/triboelectricity), it can be independently linked to one input.^[^ ^120^ ^]^ The output signal was amplified in response to the voltage bias applied at the input terminal. An ADC, depicted as “ADC” in Figure 4, transforms analog signals into digital data. This component samples the analog signal at discrete time intervals and assigns a digital value to each sample based on the signal amplitude.^[^ ^121^ ^]^ The specific circuit configuration of an ADC varies among products and is detailed in their respective datasheets. In general, ADCs require operating voltages similar to those of op‐amps, and a reference voltage that establishes the maximum range for measuring analog signals.^[^ ^122^ ^]^ The resulting digital output enhances compatibility with a wide array of signal processing, transmission, reception, and storage devices. This output was primarily demonstrated through wireless communication in standalone wearable plant‐sensing systems (Figure 4). In certain instances, signal transducers, such as the humidity sensor in Figure 4b (right), may integrate op‐amps and ADCs, whereas commercialized digital sensors often include a built‐in ADC for direct digital output.^[^ ^57^ ^]^

#### 3.2.2 Wireless Network

**Figure 5:**
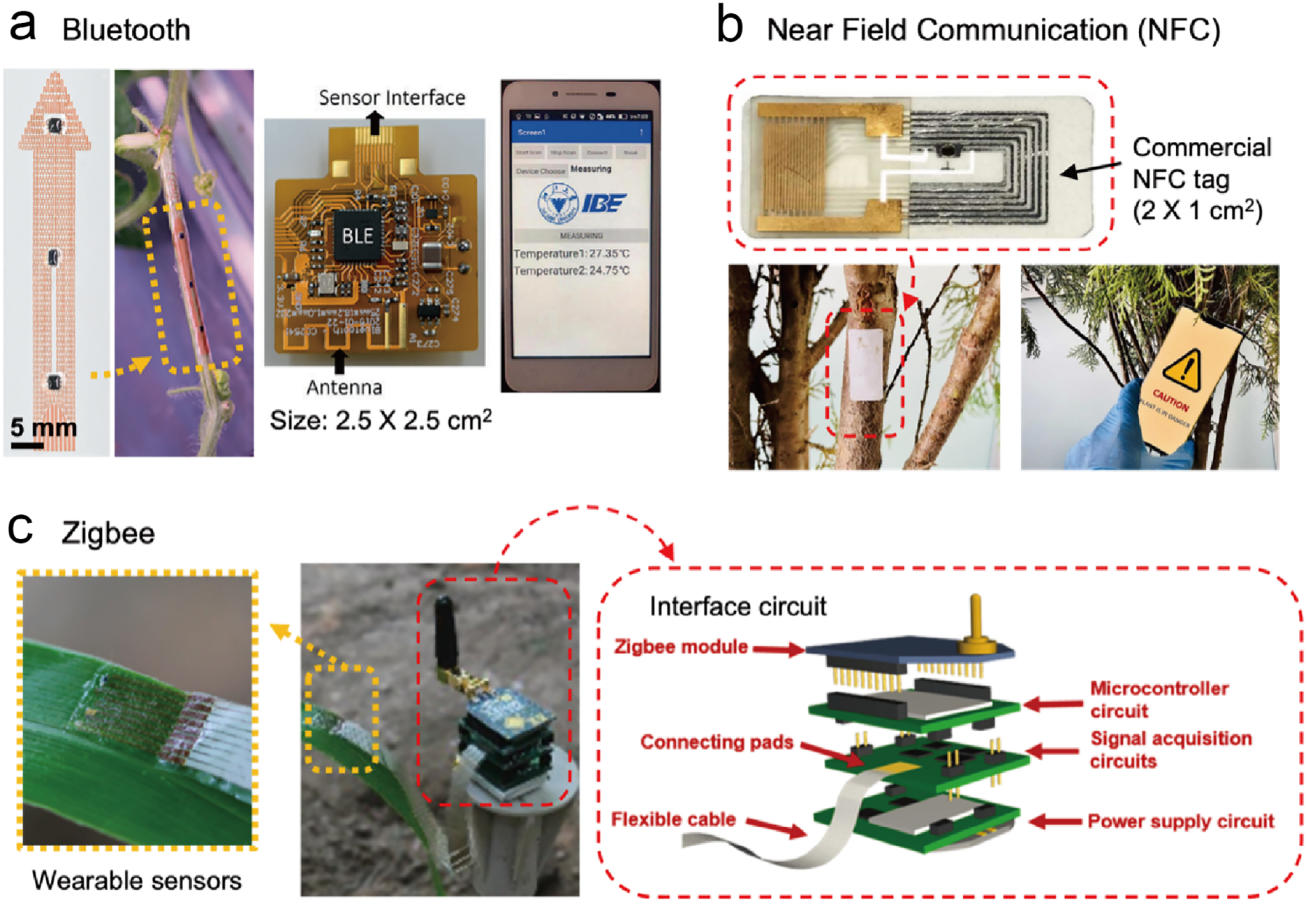
Wireless networks used in wearable plant‐sensing systems in existing studies. a) Demonstration of communication via Bluetooth of the sap flow rate monitoring performed by a wearable sensor. Reproduced with Permission.^[^ ^57^ ^]^ Copyright 2021, Wiley. b) An NFC system is used in a wearable sensor for VOC monitoring. Reproduced with Permission.^[^ ^115^ ^]^ Copyright 2023, Elsevier BV. c) Zigbee network integrated with multimodal sensors for microclimate and plant growth monitoring. Reproduced with Permission.^[^ ^116^ ^]^ Copyright 2019, American Chemical Society.

A variety of wireless networks with varying signal frequency, transmission, data rate, power consumption, and cost are available in the market. A comprehensive exploration of the characteristics of these wireless networks has been performed in smart‐farm technology reviews.^[^ ^6^, ^76^, ^123^, ^124^ ^]^ Wearable plant sensors have predominantly featured Bluetooth, Near Field Communication (NFC), or Zigbee (**Figure** **5**) owing to the familiarity of these communication technologies with the public. In this context, a comparative analysis of interface circuit configurations and wireless communication use in existing wearable standalone plant sensors is presented in this section (**Table** **1**).^[^ ^21^, ^25^, ^57^, ^58^, ^113^, ^114^, ^115^, ^116^, ^119^, ^125^, ^126^, ^127^ ^]^

**Table 1:** Comparison of wireless network, interface circuit, and sensor designs among various wearable plant sensors capable of wireless communication. The circuit size without the thickness factor indicates that the corresponding circuits are as thin as one layer of the circuit board. “†” indicates the commercial sensor used in the sensing system. “O” indicates that the system has the corresponding component, but that the model's name of the component is not reported. “‐” indicates that the corresponding information is not reported.

Network Type and Ref. [Model]	Network Type and Ref. [Model]	Op‐amps	ADC	Sensing Value	Power Consumption	Sensing Target	Data Receiver	Overall Circuit Size
Bluetooth (HJ580)^[^ ^58^ ^]^	Bluetooth (HJ580)	LM321	O	Resistance	—	Plant growth	Mobile phone	<15 × 20 mm^2^
Bluetooth (Cyble‐222005)^[^ ^21^ ^]^	Bluetooth (Cyble‐222005)^[^ ^21^ ^]^	O	O	Resistance,Capacitance	600 µA (3.7 V)	Plant growth Microclimate	Mobile phone	—
Bluetooth (CC2541)^[^ ^125^ ^]^	Bluetooth (CC2541)^[^ ^125^ ^]^	AD8606	AD5933	Resistance	—	Plant water status	Mobile phone	40 × 30 × 15 mm^3^
Bluetooth (Unreported)^[^ ^119^ ^]^	Bluetooth (Unreported)^[^ ^119^ ^]^	O	O	Capacitance	—	Plant water status	Mobile phone	—
Bluetooth (CC2541)^[^ ^57^ ^]^	Bluetooth (CC2541)^[^ ^57^ ^]^	Digital sensor	Digital sensor	Unknown (TMP102†)	—	Sap flow rate	Mobile phone	25 × 25 mm^2^, Flexible PCB
Bluetooth (EmStat3‐Blue)^[^ ^126^ ^]^	Bluetooth (EmStat3‐Blue)^[^ ^126^ ^]^	—	—	Current (EmStat3‐Blue†)	—	Pesticide	MobilePhone	10 × 6 × 3.4 cm^3^
RF resonator (VNA 3000)^[^ ^113^ ^]^	RF resonator (VNA 3000)^[^ ^113^ ^]^	—	—	Resistance	None	Ethylene (Fruit ripeness)	PC	No battery
RF resonator (EM4325)^[^ ^114^ ^]^	RF resonator (EM4325)^[^ ^114^ ^]^	‐	‐	Resonation	None	Temperature (Plant water status)	PC	43.7 × 43.8 mm^2^, No battery
NFC (Mobile phone)^[^ ^115^ ^]^	NFC (Mobile phone)^[^ ^115^ ^]^	—	‐	Resistance	None	α–pinene	Mobile phone	No battery
Zigbee (XBee‐PRO 900HP)^[^ ^116^ ^]^	Zigbee (XBee‐PRO900HP)^[^ ^116^ ^]^	O	O	Impedance, Resistance, Current	165mWh	Microclimates	PC	30 × 30 × 25 mm^3^, 4 PCB layers
Zigbee (CC2530)^[^ ^127^ ^]^	Zigbee (CC2530)^[^ ^127^ ^]^	Digitalsensor	O	Unknown (TMP36†)	30mA(3V)	Plant water status	PC	22 × 53 mm^2^
WiFi (ESP32)^[^ ^25^ ^]^	WiFi (ESP32)^[^ ^25^ ^]^	—	LTC 2421	Resistance	—	Plant pulse(Growth rate)	Mobile phone	Flexible PCB

Bluetooth, owing to its wide popularity, stands out as a prominent choice for emphasizing the user‐friendliness of plant sensors. It boasts exceptional compatibility with mobile phones and offers a straightforward application for displaying received data, emphasizing user convenience with impactful visuals (Figure 5a).^[^ ^57^ ^]^ Most interface circuits between the sensors and Bluetooth components incorporate op‐amps and ADCs (Table 1, Bluetooth section), with some using commercial sensors or devices with built‐in op‐amps or ADCs. There has been a predominant trend to employ resistance and capacitance sensors in conjunction with Bluetooth to measure plant growth and water status.^[^ ^21^, ^57^, ^58^, ^119^, ^125^ ^]^ Efforts have also been made to enhance the wearability of commercial sensing devices with Bluetooth capabilities through the development of stretchable electrodes.^[^ ^126^ ^]^

Radio frequency (RF) resonation, including NFC, is characterized by a shorter network distance than Bluetooth^[^ ^123^ ^]^ and is familiar to customers owing to everyday card‐tapping payment systems. In this approach, interface circuits do not require op‐amps, ADCs, or even batteries since they adopt a straightforward design with coil‐shaped signal transmitters (Table 1, RF resonator and NFC section).^[^ ^113^, ^114^, ^115^ ^]^ This network type ensures that sensors exhibit high wearability, minimizing physical and chemical interference with plants. Several studies have demonstrated the ease of observing plant‐related gases (Figure 5b)^[^ ^115^ ^]^ and temperatures using mobile phones or dedicated receivers equipped with signal processing and power functions (Table 1, RF resonator, and NFC section). However, the limited network range poses challenges for remotely collecting sensing data from multiple points in expansive smart‐farm settings. Zigbee, although less popular among consumer devices, is actively used as a wireless network in industrial and agricultural applications.^[^ ^124^ ^]^ It has a similar or longer transmission range relative to Bluetooth and low power consumption.^[^ ^123^ ^]^ Zigbee supports large‐scale sensor networks characterized by a mesh network topology, in contrast to Bluetooth's point‐to‐point arrangement.^[^ ^128^ ^]^ Thus, Zigbee serves as an appropriate tool for managing the numerous wearable sensors tasked with ongoing plant monitoring in remote regions.^[^ ^124^ ^]^ Leveraging these advantages, a research group demonstrated the feasibility of a wearable standalone system featuring multimodal sensors for microclimate and plant growth monitoring (Figure 5c).^[^ ^116^ ^]^ However, the use of Zigbee in wearable sensors has been shown to be limited in the existing literature (Table 1, Zigbee section).^[^ ^116^, ^127^ ^]^ In the broader context of smart‐farm technology, diverse network types such as Zigbee, WiFi, 3G/4G, SigFox, NB‐IoT, and LoRa are employed for large‐scale plant sensor management across expansive areas.^[^ ^6^ ^]^ Consequently, in alignment with the overall trajectory of smart‐farm technology, it is imperative to actively explore a variety of networks in wearable plant sensors to demonstrate their commercial potential.

#### 3.2.3 In Situ Monitoring

**Figure 6:**
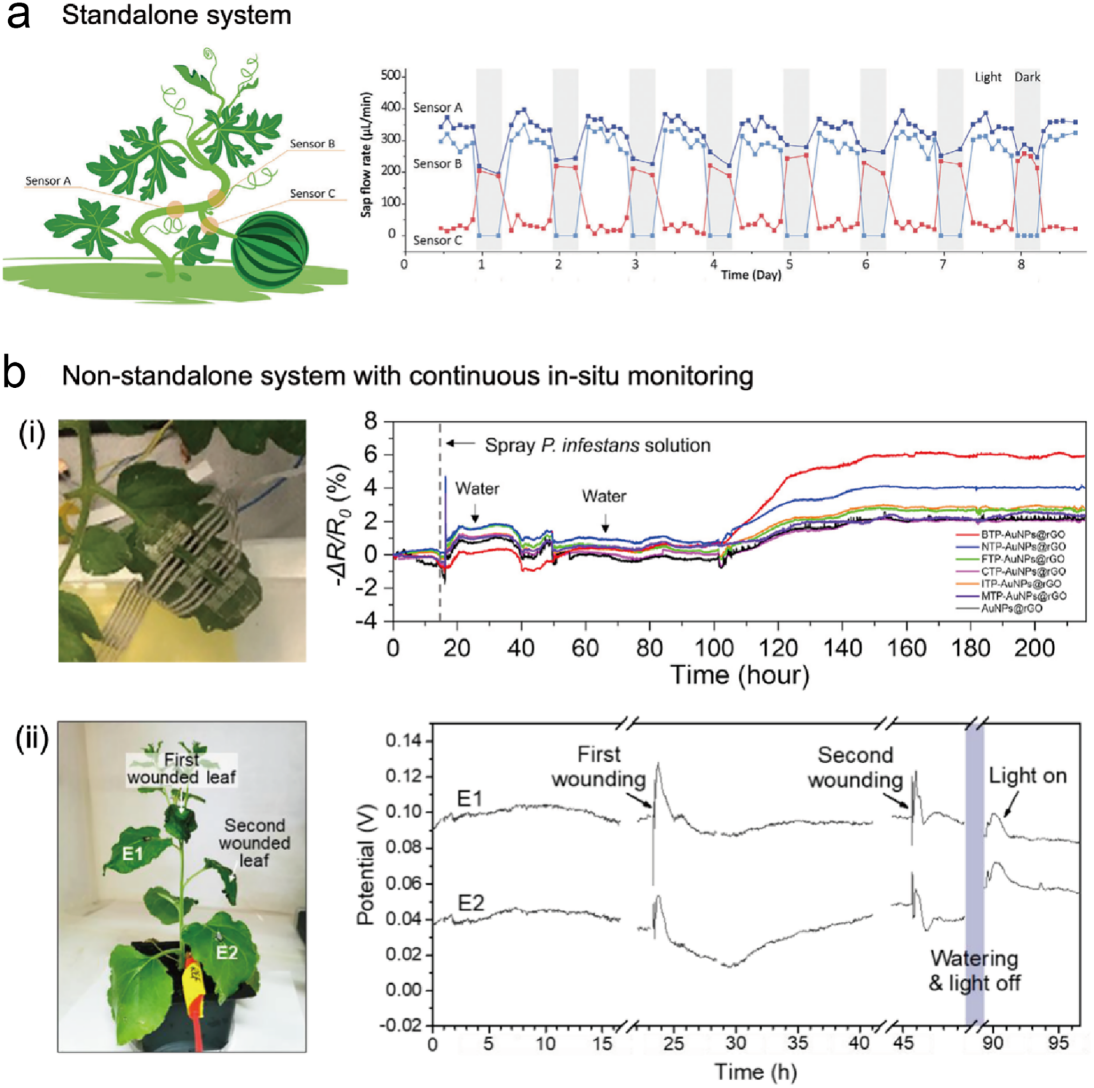
In situ monitoring of standalone and non‐standalone wearable sensing systems a) Sap flow rate monitoring for a watermelon plant with a standalone system. Reproduced with Permission.^[^ ^57^ ^]^ Copyright 2021, Wiley. b) Continuous monitoring via a non‐standalone system for i) pathogen inoculation and plant water status and ii) physical wound and abiotic stress. i) and ii) Reproduced with Permission, respectively.^[^ ^19^, ^131^ ^]^ Copyright 2021, Elsevier BV, and Copyright 2021, Wiley, respectively.

In situ (real‐time) monitoring is the primary application of wearable standalone sensors. It empowers growers and researchers to access current information about plants and their surroundings, facilitating timely decision‐making and responsive actions in the face of dynamic conditions.^[^ ^6^ ^]^ This proactive approach mitigates issues such as diseases, pests, or nutrient deficiencies while optimizing resource utilization (e.g., water, fertilizers, and energy), ultimately saving time, cost, and labor. Consequently, nearly all wireless network‐based wearable plant sensors perform in situ monitoring (**Table** **2**),^[^ ^21^, ^25^, ^57^, ^58^, ^113^, ^115^, ^116^, ^119^, ^125^, ^127^, ^129^ ^]^ with monitoring periods spanning from 1 day to over 1–2 months, primarily exceeding 1 week. Data pertaining to plant growth,^[^ ^21^, ^25^, ^58^ ^]^ microclimate parameters, such as temperature and humidity,^[^ ^116^ ^]^ and vital VOCs were collected for assessing plant health and environmental conditions.^[^ ^113^, ^115^ ^]^ Furthermore, crop quality in plants such as soy, melon, and watermelon can be assessed both qualitatively and quantitatively by observing their water status.^[^ ^57^, ^125^, ^127^ ^]^ For instance, in one study,^[^ ^57^ ^]^ three wearable plant‐sensing systems, each equipped with two temperature sensors, were affixed around a watermelon fruit (**Figure** **6**a) to monitor sap flow rates. The findings showed variations in sap flow toward the fruit depending on day and night and the cessation of sap flow in response to physical damage.

**Table 2:** Comparison of the in situ monitoring design among wearable standalone plant‐sensing systems. ‘†’ indicates the commercial sensor used in the sensing system. “VOC” indicates a volatile organic compound.

Sensing Target and Ref.	Sensing Value	Duration	Temporal Resolution	Plant
Plant growth^[^ ^58^ ^]^	Resistance	5 days	1 h	Bamboo
Plant growth^[^ ^21^ ^]^	Resistance	2 days	Many/min	Bamboo
Plant growth^[^ ^25^ ^]^	Resistance	11 days	≈30 h	Tomato plants
Microclimates^[^ ^116^ ^]^	Impedance, Resistance, Current	2 days	Continuous	*Scindapsus aureus*
Ethylene (Fruit ripeness)^[^ ^113^ ^]^	Resistance	8 days	1/day	Climacteric fruits
α–pinene (VOC)^[^ ^115^ ^]^	Resistance	45 days	Several/10h	*Platanus orientalis*
Electrochemical potential^[^ ^129^ ^]^	Voltage	>7 days	1 s or 1 min	Potted/grounded plants
Plant water status^[^ ^125^ ^]^	Resistance	1 day	1 h	Soy Plants
Plant water status (Δ Temperature)^[^ ^127^ ^]^	Unknown (TMP36†)	>2 months	1/day	Melon plants
Plant water status (Δ Stoma size)^[^ ^119^ ^]^	Capacitance	>12 days	2/day	*Nicotiana tabacum*
Sap flow rate (Δ Temperature)^[^ ^57^ ^]^	Unknown (TMP102†)	>16 days	9/day	Watermelon plants

Standalone sensors exhibit limited temporal resolution, as evident in the monitoring results depicted in Figure 6a (right). This limitation may stem from factors such as transmission/reception cycles and data storage. Some measurements are relatively continuous, occurring at minute intervals, however, most operate with sampling rates spanning hours or even days (Table 2, Temporal resolution), falling short of the sampling frequency expected for in situ monitoring, hence, they struggle to capture sudden plant stress or environmental shifts. Conversely, there exist many non‐standalone systems with notably high temporal resolution (**Table** **3**).^[^ ^9^, ^19^, ^20^, ^55^, ^93^, ^130^, ^131^, ^132^, ^133^, ^134^ ^]^ These systems commonly conduct measurements over several days or longer (Table 3, Duration), boasting rapid sampling rates that render monitored values as continuous lines. This enhanced temporal resolution not only enables more precise observations of microclimate and plant water status (Figure 6b(i)),^[^ ^19^ ^]^ but also facilitates the detection of biotic stress (Figure 6b,(i)) and abrupt physical or abiotic stressors (Figure 6b(ii)).^[^ ^131^ ^]^ High temporal resolution and precise measurements have been achieved because of the use of substantial analytical equipment, which is bulky in most cases. These findings underscore the need for standalone systems to evolve toward providing more accurate and extended observations, similar to those provided by non‐standalone systems.

**Table 3:** Comparison of the in situ monitoring design among non‐standalone wearable plant‐sensing systems. All systems in this table showed continuous real‐time monitoring with high temporal resolution, different from the standalone systems summarized in Table 2.

Sensing Target and Ref.	Sensing Value	Duration	Plant
Temperature, humidity, light^[^ ^20^ ^]^	Resistance	>15 days	Money tree
VOC, temperature, humidity^[^ ^9^ ^]^	Resistance, Capacitance	14 days	Tomato plant
Nitrate uptake^[^ ^130^ ^]^	(Drain) Current	>7 days	Corn plants
Biotic stresses, Plant water status^[^ ^19^ ^]^	Resistance	≈4 days	Tomato plant
Physical wound^[^ ^131^ ^]^	Electrical potential	>4 days	tobacco plants
Plant water status^[^ ^93^ ^]^	Resistance	7 days	Peace Lily
Plant water status^[^ ^132^ ^]^	Capacitance	>11 days	Tomato plant
Plant water status^[^ ^133^ ^]^	Electrical potential	>12 days	Tomato plant
Plant water status^[^ ^134^ ^]^	Electrical potential	>11 days	Aloe leaf
Solute content of the plant sap^[^ ^55^ ^]^	(Drain) Current	>22 days	Tomato plant

#### 3.2.4 Data Manipulation

**Figure 7:**
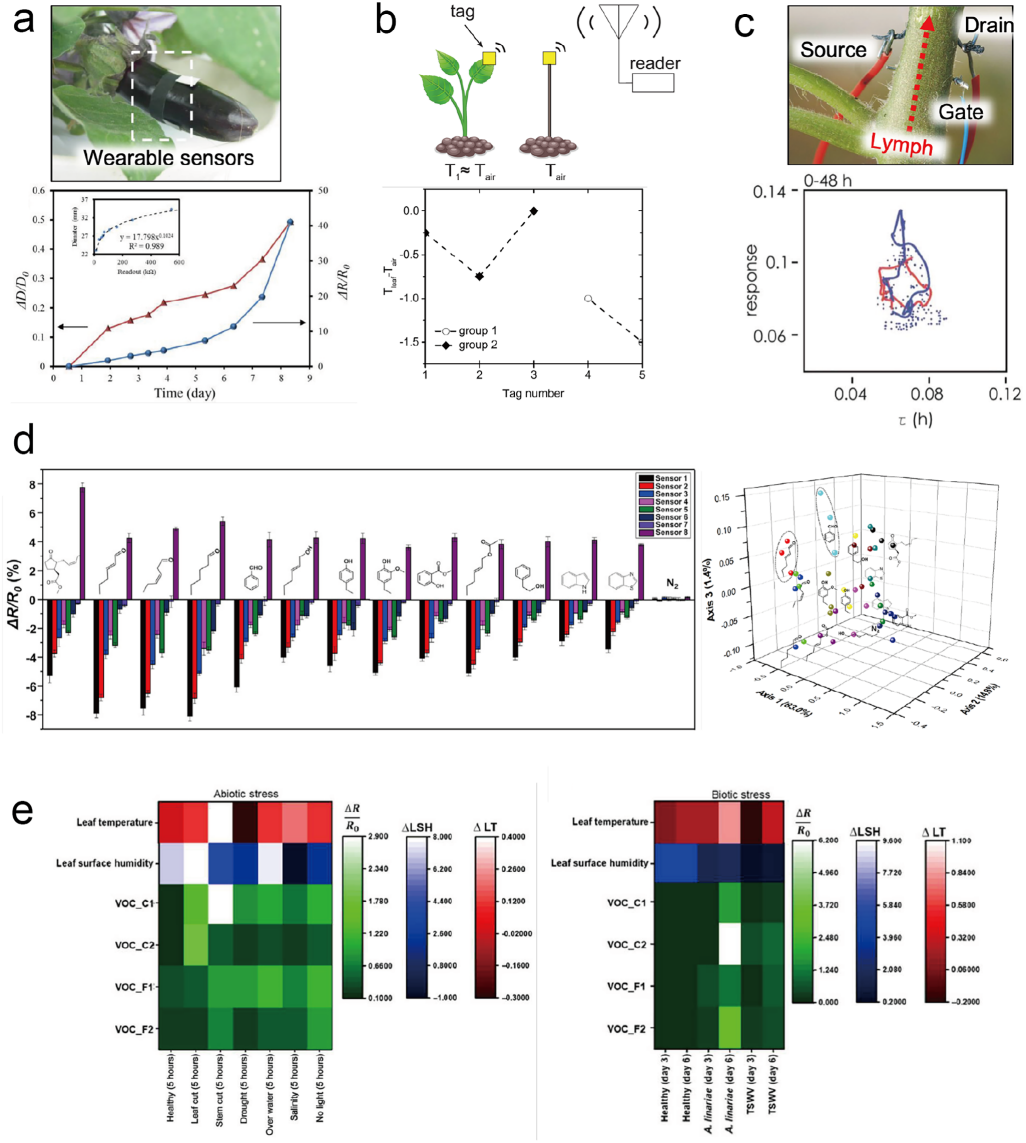
Data manipulation in a wearable (non‐standalone) plant‐sensing system. a) Fruit diameter (*D*) measurement based on an empirical formula (inset) related to the resistance value. Reproduced with Permission.^[^ ^59^ ^]^ Copyright 2019, Elsevier BV. b) Plant water status measurement is based on the temperature difference between a leaf (*T*_leaf_) and air (*T*_air_). Reproduced with Permission.^[^ ^114^ ^]^ Copyright 2019, Institute of Electrical and Electronics Engineers. c) Physiological plant status monitoring is based on two factors reflecting the ionic composition of plant sap (that is, time constant, *τ*) and the number of ions collected within a given time interval (that is, response). Reproduced with Permission.^[^ ^55^ ^]^ Copyright 2017, Nature Publishing Group. d) Discrimination of individual plant VOCs based on the sensing results of eight resistance sensors. Reproduced with Permission.^[^ ^19^ ^]^ Copyright 2021, Elsevier BV. e) Heatmaps of sensor data after abiotic (left) and biotic (right) stresses. Reproduced with Permission.^[^ ^9^ ^]^ Copyright 2023, American Association for the Advancement of Science.

Most existing wearable standalone systems primarily focus on transmitting raw electrical signal data (e.g., resistance, capacitance, and current) acquired from sensors to mobile devices or personal computers (PCs) (Table 2). However, enhancing the interface circuit's capabilities to perform data manipulation and visualization functions, as demonstrated in some non‐standalone systems,^[^ ^9^, ^19^, ^55^, ^59^, ^114^ ^]^ can greatly assist users in interpreting sensor data. It empowers growers and users to make informed and expedited decisions regarding plant management. This section outlines the direction of data manipulation in standalone systems, drawing insights from various examples of non‐standalone systems. For instance, when employing a resistance sensor wrapped around a fruit, an empirical formula for correlating changes in the fruit diameter with resistance changes can be developed and integrated into the sensor interface (**Figure** **7**a).^[^ ^59^ ^]^ This enables users to intuitively gage the fruit size when using the sensor. Moreover, the temperature difference between leaves and the surrounding air can be harnessed to indirectly assess plant water status;^[^ ^135^ ^]^ two wearable temperature sensors can be employed to promptly convey this information to users (Figure 7b).^[^ ^114^ ^]^ If the interface circuit can visualize the results of monitoring ion count and composition changes in the plant sap using electrochemical sensors (Figure 7c),^[^ ^55^ ^]^ it offers an intuitive understanding of the plant's physiological status.^[^ ^136^, ^137^ ^]^ Another study presented a method employing eight sensors for visually classifying 13 different VOCs on a three‐axis graph, achieving over 95% discrimination accuracy for individual plant VOCs (Figure 7d).^[^ ^19^ ^]^ Embedding the logic of this classification method within the interface circuit could significantly enhance VOC detection efficiency. Additionally, if the interface circuit can display the simultaneous monitoring results of leaf temperature, humidity, or key VOCs as a heatmap (Figure 7e),^[^ ^9^ ^]^ users will gain a more convenient method for identifying the occurrence and impact of biotic and abiotic stress on plants.

### 3.3 Power Supplies

Standalone wearable sensing systems for plant monitoring require a sustainable power supply for continuous operation.^[^ ^138^ ^]^ The repetitive replacement of batteries or the presence of numerous long wires to connect to power sources located far from the plants not only complicates plant wearable systems but also makes them impractical in terms of labor and cost.^[^ ^139^ ^]^ Therefore, harvesting energy from the plant itself or from the environment surrounding the plant and converting it into electrical energy is the most effective way to supply electrical power to plant wearable systems. In this section, we briefly review the representative energy‐harvesting technologies suitable for self‐powered plant wearables, including triboelectric nanogenerators and biofuel cells.

#### 3.3.1 Triboelectric Nanogenerators

A TENG is an energy generator that converts mechanical energy into electrical power. Conversion occurs via triboelectrification, a process wherein electric charges are transferred between two objects when they come into contact with each other via mechanical processes. When two triboelectric materials are in contact, contact electrification results in the formation of surface charges on both materials. The separation of the two materials subsequently leads to the localization of opposite charges on both surfaces, causing an electric potential difference between the two materials. This potential difference initiates the flow of current in an electric circuit via electrostatic induction, thus generating electric power. Various materials and device structures have been studied to enhance the electric power output of TENGs, and numerous applications of TENGs have been explored.^[^ ^140^, ^141^, ^142^, ^143^ ^]^ TENGs are a promising candidate as the power supply generator of wearable standalone electronic systems for plants because plants can provide the mechanical movements required by TENGs through the movement caused by wind and rainfall droplets, which are omnipresent in their growth environment. In this regard, several studies have attempted to employ TENGs with plants and subsequently harvest electrical energy from the movements of plants.^[^ ^143^, ^144^, ^145^, ^146^ ^]^ Fortunately, plants often exhibit sophisticated intrinsic surface microstructures that are beneficial for triboelectric energy conversion because they provide an enlarged contact area and increased friction.^[^ ^143^, ^147^ ^]^

**Figure 8:**
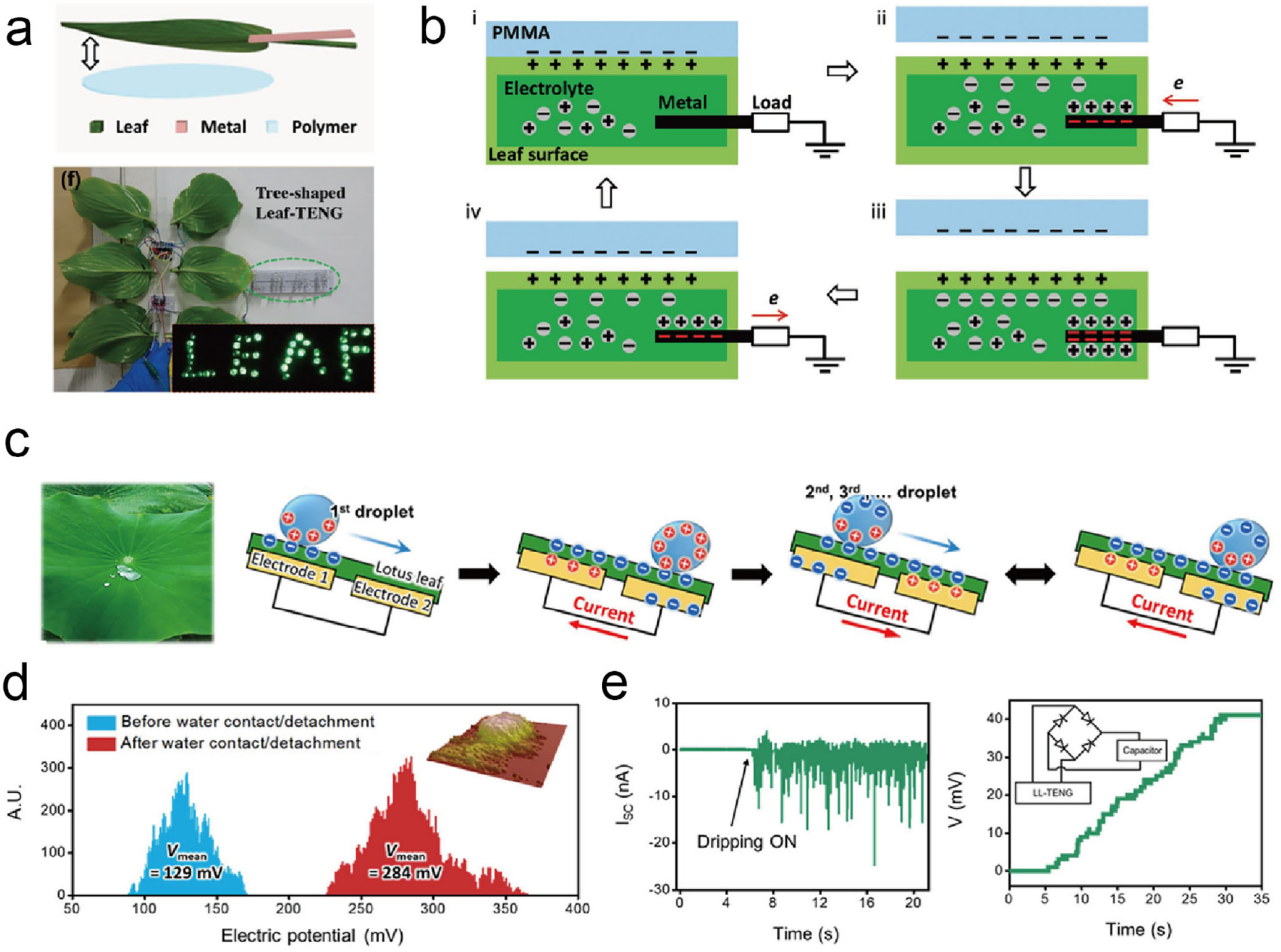
The structure and operation of the leaf‐assembled TENG. a) Schematic of Leaf‐TENG. b) Illustration of the working principles of Leaf‐TENG in the single electrode mode: i) contact state, ii) separating state, iii) separated state, iv) approaching state. c) Image of the natural lotus leaf with a rolling water droplet on its surface (left) and the operation mechanism of the lotus leaf (LL)‐TENG that possesses two strips of the Au electrode (right). d) Measurement of the electric potential before and after sequential contact and detachment of the water droplet on the lotus leaf. e) Short‐circuit current (I_SC_) generated from the LL‐TENG with falling water droplets on its surface (left) and charging of a conventional capacitor connected to the LL‐TENG (right). Figure (a–e) Reproduced with Permission^[^ ^148^ ^]^ and,^[^ ^152^ ^]^ respectively. Copyright 2018, Wiley and Copyright 2017, Elsevier BV.

The first study that demonstrated plant‐integrated TENG for mechanical energy harvesting was conducted by Y. Jie et al.^[^ ^148^ ^]^ In this study, the TENG was assembled on a natural leaf of *Hosta plantaginea* (**Figure** **8**a). The natural leaf itself was used as the triboelectric layer and electrode, and a metal electrode was connected to the leaf for an electrical connection. A sheet of poly (methyl methacrylate) (PMMA) was used as the contact layer. When the leaf was in contact with the PMMA sheet via an external mechanical force (Figure 8b), electrification occurred between the leaf surface and the PMMA sheet owing to the difference in their electron affinity. After separating the PMMA sheet from the leaf, the positive charges on the leaf surface induced the movement of ions within the leaf, together with the formation of an electrical double layer at the metal‐leaf electrolyte interface. Consequently, electron flow occurred in the external circuit. This leaf‐TENG exhibited a maximum output power density of 45 mW m^−2^ under optimized conditions with a contact area of 64 cm^2^, a mechanical movement frequency of 2 Hz, a peak velocity of 0.333 m s^−1^, and an external resistance of 10 MΩ. The authors found that various natural leaves other than *Hosta plantaginea* leaves can also be used to fabricate leaf‐TENG, however, it must be noted that the electrical output of the leaf‐TENG showed considerable dependence on the leaf species because of their different electron affinities and surface microstructures. Later, F. Meder et al. also reported similar results using *Rhododendron* leaves, achieving a maximum output power density of 150 mW m^−2^ under the following conditions: A contact area of 25 mm^2^ of the Ecoflex pad, an impact force of 0.9 N, mechanical frequency of 10 Hz, and external resistance of 200–300 MΩ.^[^ ^149^ ^]^ Highly flexible TENGs that can be attached to plant leaves have also been demonstrated in previous studies. L. Lan et al. developed a highly flexible and stretchable TENG by encapsulating a silver nanowire (AgNW) and metallic MoS_2_ nanosheet‐based conductive composite film with two layers of PDMS.^[^ ^150^ ^]^ The device showed excellent flexibility and stretchability and could be attached conformally to plant leaves despite their nonplanar and irregular shapes. The PDMS encapsulation layer and AgNW‐MoS_2_ conducting film served as the triboelectric layer and electrode of the TENG, respectively. The TENG exhibited a maximum power density of 160 mW m^−2^ under the following conditions: 250 mm^2^ contact area with skin/leaf, impact force of 10 N, mechanical frequency of 10 Hz, and external resistance of 40 MΩ. Similarly, C. Jiang et al. reported a flexible leaf‐attachable TENG based on a porous PDMS‐MXene composite film and a laser‐induced graphene electrode.^[^ ^151^ ^]^ The incorporation of MXene enhanced the triboelectronegativity of the PDMS film, resulting in a voltage output seven times higher for PDMS‐MXene‐based TENG than that for pure PDMS‐based TENG. Later, L. Lan et al. developed a leaf‐attachable TENG that showed improved performance by using CNTs as the electrode and fluorinated CNT microsphere‐embedded poly (vinylidene fluoride‐co‐hexafluoropropylene) (PVDF‐HFP) nanofiber film as the triboelectric layer.^[^ ^139^ ^]^ This TENG exhibited a maximum power density of 3306 mW m^−2^ for a device with a contact area of 400 mm^2^ at a mechanical frequency of 5 Hz, impact force of 30 N, and an external resistance of 10 MΩ. Additionally, this TENG did not affect the intrinsic physiological activities of the leaves, although it was conformally attached to the leaves (i.e., the TENG exhibited “breathability”), as it had an inherent porous nanofiber network structure that allowed efficient permeation of water vapor.

In addition to solid‐solid contact electrification, liquid‐solid contact electrification can be used to convert mechanical energy applied to plants. D. Choi et al. demonstrated a lotus leaf‐based TENG that used discrete liquid‐solid contact electrification, it showed the consequent generation of electrical charges on natural lotus leaves (Figure 8c,d).^[^ ^152^ ^]^ Discrete liquid‐solid contact electrification is a process wherein sequential contact and detachment of an aqueous liquid from a solid surface result in the generation of net electrical charges on both the liquid and solid surfaces. When water droplets fell onto the surface of lotus leaves, they rolled on the surface without wetting the leaves because of the hydrophobicity of the leaves’ surface. The rolling of water droplets could be considered sequential contact and detachment of water droplets from the leaf surface, thus leading to the generation of surface charges on the leaf by the occurrence of discrete liquid‐solid contact electrification. By simply depositing a metal electrode on the reverse side of the natural lotus leaf surface, a lotus leaf‐based TENG was fabricated (Figure 8e). The maximum output power density of the lotus‐leaf‐based TENG was 2.5 µW cm^−2^ when a 30 µL deionized water droplet was dropped onto the TENG at an external resistance of 100 MΩ. H. Wu et al. also demonstrated a TENG based on water droplets and natural leaves.^[^ ^153^ ^]^ They used a leaf of *Mytilaria laosensis*, wherein the ion‐conductive tissue and plant cuticle of the leaf served as the electrode and triboelectric active material, respectively. The TENG exhibited a maximum output power density of tens of mW m^−2^ at the external resistance of 200 kΩ when 33 µL of 100 mM NaCl solution droplets impacted the leaf surface.

#### 3.3.2 Biofuel Cells

A biofuel cell is a device that converts the chemical energy of a molecule within a biofluid into electrical energy via bioelectrochemical reactions occurring at the cathode and anode. In a biofuel cell, one or both electrodes can be an enzymatic electrode, in an enzymatic electrode, an enzyme is immobilized at the electrode to promote the redox reaction of the biofuel.^[^ ^154^, ^155^ ^]^ The operation of a biofuel cell is similar to that of a conventional fuel cell, except for some reaction steps involving enzymes.^[^ ^155^, ^156^ ^]^ When a metabolite in a biofluid (e.g., glucose) is oxidized at the anode of the biofuel cell, electrons are transferred to the anode and flow through the external circuit to reach the cathode of the biofuel cell. Subsequently, the electrons are transferred to an oxidant, usually oxygen molecules, within the biofluid at the cathode surface, this reduces the oxidant and completes the pair of electrochemical redox reactions. Because electrical power is generated by these redox reactions, its magnitude strongly depends on the chemical species used as biofuel and their concentration within the biofluid. Additionally, the performance of biofuel cells depends on several other factors that affect the rate of redox reactions, such as temperature, pH, humidity, oxygen levels, and the presence of other substances within the biofluid.^[^ ^155^, ^157^ ^]^ Therefore, careful consideration of the selection of biofuel and enzymes, as well as the environment of plant growth, is required to increase the performance of biofuel cells for plant wearable applications.^[^ ^157^, ^158^, ^159^ ^]^

**Figure 9:**
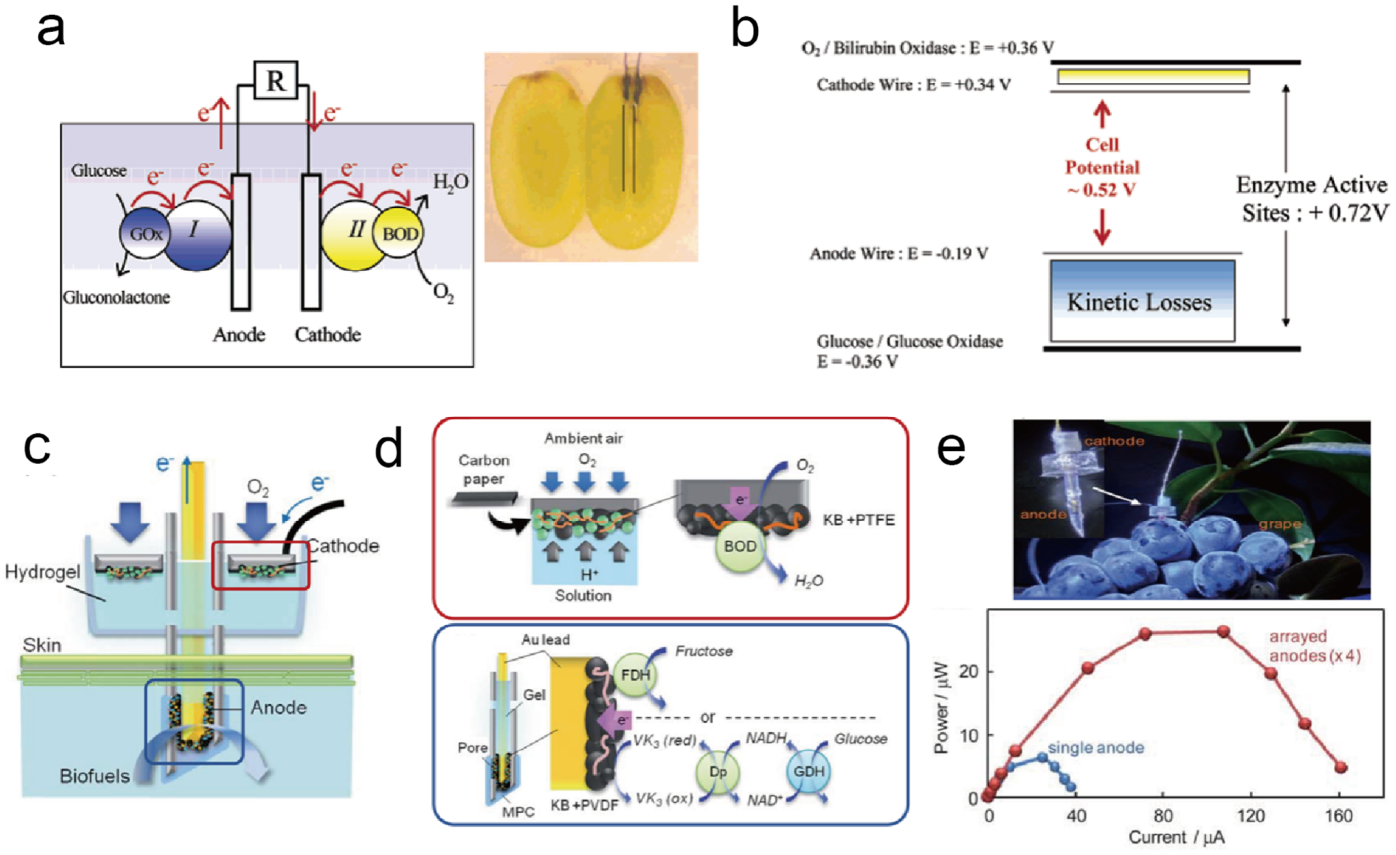
The scheme of biofuel cells inserted into a living organism. a) Diagram of a compartment‐less biofuel cell (left) and a photograph of a sliced grape with implanted fibers and their electrical contacts. b) Operating potentials (vs. Ag/AgCl at pH 7.2) of enzymes and their “wiring” redox polymers. c) Schematic illustration of the structure of a biofuel cell with a needle‐shaped anode and a gas‐diffusion cathode. d) Schemes of O_2_ reduction at the enzymatic gas‐diffusion cathode (top) and schemes of fructose and glucose oxidation at the enzymatic needle anodes (bottom). e) Photograph of the assembled biofuel cell (with needle‐shaped anode and gas‐diffusion cathode) inserted into a grape (top) and the power output of the biofuel cell by using a single anode and four arrayed anodes (bottom). Figure (a–e) Reproduced with Permission^[^ ^157^ ^]^ and,^[^ ^160^ ^]^ respectively. Copyright 2003, American Chemical Society and Copyright 2011, Royal Society of Chemistry.

In the case of plants, the most abundant biofuels are sugars such as glucose and fructose, hence, these sugars are widely used for biofuel cells using plants.^[^ ^138^, ^154^ ^]^ N. Mano et al. demonstrated the operation of a glucose‐O_2_ biofuel cell in a living grape.^[^ ^157^ ^]^ In this study, the biofuel cell comprised two enzyme‐coated carbon fibers with a 7 µm diameter and 2 cm length (**Figure** **9**a). The anodic enzyme glucose oxidase was electrostatically attached to a polymer (polymer I) on the anode side, and the cathodic enzyme bilirubin oxidase was attached to another polymer (polymer II) on the cathode side. This structure provided an energetic cascade of electrons in the following order: glucose, glucose oxidase, polymer I, anode, external resistance, cathode, polymer II, bilirubin oxidase, and O_2_. Implantation of the electrodes in a grape with a glucose concentration higher than 30 mM and pH 5.4, yielded a maximum power output of 2400 mW m^−2^ with a cell voltage of 0.52 V (Figure 9b). T. Miyake et al. also demonstrated the operation of a biofuel cell in grapes.^[^ ^160^ ^]^ The authors attempted to develop a more practical form of a cell that could be easily applied to natural plants with skin. To achieve this, they fabricated a biofuel cell with a novel structure consisting of a needle‐shaped anode and a gas‐diffusion cathode (Figure 9c). The needle‐shaped anode was inserted into a grape for the oxidation of fructose or glucose inside the fruit (Figure 9d, top), whereas the cathode was placed in ambient air outside the grape skin to reduce oxygen in the air (Figure 9d, bottom). This structure not only allowed easy insertion of the anode through plant skins but also provided the cathode with an environment in which the cathode reaction was not disturbed by other molecules within the biofluids and was supplied with sufficient oxygen. The cell using grape exhibited a maximum output power of 1150 mW m^−2^ with a cell current of 25 µA and a cell voltage of 0.25 V (Figure 9e). V. Flexer et al. studied the effects of photosynthesis on the performance of biofuel cells in plants.^[^ ^159^ ^]^ They found that the power density of glucose‐O_2_ biofuel cells fabricated using a cactus plant was strongly dependent on the presence of light. When the cactus plant was illuminated, a maximum power density of 90 mW m^−2^ was obtained at a cell voltage of 0.4 V, which was ≈70% higher than that obtained from the cactus plant placed in the dark. The authors attributed this increase to higher concentrations of glucose and O_2_ in the illuminated cactus, which are the products of photosynthesis.

#### 3.3.3 Limitations and Challenges

The power required for operating wearable sensing systems, which consist of multiple components such as sensors, data processing modules, and wireless transmission units, typically ranges from a few milliwatts to several watts.^[^ ^161^, ^162^, ^163^, ^164^ ^]^ The average power consumption of these systems can be significantly reduced when sensors operate at low sampling rates or when data transmission occurs intermittently. In contrast, the reported maximum power output densities of TENGs and biofuel cells (BFCs) fall within the range of 0.1 µW cm^−2^ to a few mW cm^−2^.^[^ ^156^, ^165^ ^]^This indicates that the power generated by these energy sources can either meet or fall short of the requirements of wearable sensing systems, depending on their operational characteristics. Specifically, TENGs and BFCs may provide sufficient power for low‐frequency sensing tasks and intermittent data transmission but are likely inadequate for applications requiring real‐time sensing and continuous wireless data transmission. Another critical limitation of these energy sources is their instability, as their power output is highly dependent on environmental conditions, while electronic devices generally require a stable and consistent power supply. Therefore, integrating TENGs or BFCs with power management circuits or energy storage units is essential to achieve a sustainable power supply for plant‐wearable sensing systems. Considerable research efforts have focused on improving the power output of TENGs and BFCs and ensuring their stability as reliable and sustainable energy sources,^[^ ^165^, ^166^, ^167^, ^168^ ^]^ and these endeavors remain a pivotal area of research for advancing the development of stand‐alone plant‐wearable sensing systems.

The degradability of electronic components is another crucial factor in the development of plant‐wearable sensing systems. By utilizing materials that naturally decompose into non‐toxic byproducts, the long‐term environmental impact of electronic waste can be minimized. This is particularly important in plant‐wearable electronics, as retrieving deployed devices is often not economically feasible, and consequently, these devices are typically expected to be left in place even after their operational lifespan has ended. For TENGs, degradability can be achieved through the use of biodegradable triboelectric layer materials such as chitosan, cellulose, silk fibroin, alginate, PLA, polycaprolactone (PCL), and polyvinyl alcohol (PVA).^[^ ^169^, ^170^ ^]^ Since their introduction in TENGs, these materials have been optimized to enhance durability and triboelectric performance, making them suitable for energy generation applications. For instance, Y. Quan et al. recently investigated the improvement of mechanical and triboelectric properties of PVA‐based porous films by incorporating *β*‐lactoglobulin fibrils. The resulting porous film exhibited significantly increased fracture strength and flexibility, along with enhanced triboelectric performance.^[^ ^171^ ^]^ Despite substantial advancements in degradable TENGs in recent years, several challenges remain for their practical application. First, the power output of degradable TENGs must be further improved to enable more effective energy harvesting. Additionally, controlling the degradation rate of materials is another critical issue. If degradation occurs too rapidly, the device's lifespan and reliability would be severely limited. Therefore, strategies to precisely regulate the material degradation rates in degradable TENGs must be developed. Finally, achieving fully degradable TENGs is essential for environmental sustainability, as many current degradable TENGs still contain non‐degradable components.^[^ ^170^ ^]^ In the case of BFCs, they are generally regarded as having a low environmental impact due to their use of eco‐friendly biocatalysts and biofuels, which are inherently biodegradable.^[^ ^168^, ^172^ ^]^ Paper‐based BFCs serve as a representative example of fully disposable and biodegradable BFCs.^[^ ^173^, ^174^ ^]^

Finally, we briefly discuss the economic feasibility of TENGs and BFCs as power sources for plant‐wearable sensing systems. To be viable for large‐scale and disposable applications, these power sources must be available at a low cost, otherwise, their commercial adoption would be impractical. The cost of triboelectric materials is a critical factor influencing the overall expense of TENGs. Numerous studies have emphasized the importance of utilizing inexpensive, abundant, and recyclable materials to reduce the production costs of TENGs. For instance, overhead projector sheets have been incorporated into a TENG as a cost‐effective alternative to relatively expensive materials such as polytetrafluoroethylene and PDMS.^[^ ^175^ ^]^ Additionally, natural cellulose, which is significantly more affordable than traditional fluorinated polymers, can be chemically modified to enhance its triboelectric properties, making it a promising material for TENG fabrication.^[^ ^176^ ^]^Waste materials, such as milk cartons and aluminum packaging, can also repurposed as triboelectric layers, effectively eliminating raw material costs.^[^ ^176^ ^]^ Consequently, active research efforts are being conducted to develop cost‐effective TENGs, with promising results achieved through the use of low‐cost materials and recycled waste. For BFCs, the primary obstacle to cost reduction lies in the expensive materials required for electrodes and catalysts. In particular, enzymatic biofuel cells involve multiple complex and labor‐intensive steps, including enzyme extraction, isolation, purification, stabilization, and immobilization, all of which contribute significantly to production costs.^[^ ^172^ ^]^Currently, the cost‐performance ratio of enzymatic biofuel cells is not competitive with other energy technologies due to their low power generation. Moreover, their unstable enzymatic activity and susceptibility to denaturation prevent them from long‐term and stable power generation, further increasing operation costs.^[^ ^168^, ^172^ ^]^Therefore, enhancing the electrical performance and extending the operational lifespan of BFCs are key challenges that must be addressed to achieve their commercial viability.

### 3.4 Machine Learning in Wearable Plant Sensors

AI has spurred innovation in various fields of science, with deep learning (DL) algorithms at the forefront of their widespread applications.^[^ ^177^, ^178^ ^]^ These algorithms, a subset of ML, have made remarkable advances in fields such as computer vision, natural language processing, sensors, human‐machine interfaces, and robotics. Intelligent wearable sensing systems, distinct from their conventional counterparts, excel at collecting, interpreting, accessing, and analyzing data as well as facilitating adaptation to their environments by providing feedback signals. This process relies heavily on sensor devices that collect data, that are subsequently processed using ML algorithms. With the proliferation of technologies such as IoT and data storage systems, which generate large and complex datasets, ML algorithms have become essential for identifying trends and making predictions within these datasets, thereby enhancing the capabilities of intelligent sensing platforms. However, acquiring high‐quality sensor data remains a challenge because of the complex and irregular nature of the data. In various fields, multifunctional wearable devices are attracting increasing attention, particularly in healthcare, electronic skins, wearable electronics, and robotics.^[^ ^17^, ^100^, ^179^ ^]^ Wearable sensors are crucial for obtaining high‐quality data, and the integration of AI with flexible electronics has led to the development of intelligent wearable sensing systems that can be used as plant‐monitoring devices.^[^ ^180^ ^]^ ML algorithms have emerged as critical tools for analyzing the diverse types of electrical signals generated by wearable sensing systems, such as voltage, current, charge, and capacitance, thereby improving decision‐making accuracy. These systems primarily rely on the collection of raw sensor data, which can take various forms, including discrete data, time series data, mapping data, and data derived from multiple sensors. Time series and mapping data are particularly favorable for intelligent wearable systems. However, to be compatible with ML algorithms, the collected data requires preparation, including data cleaning, curation, and feature extraction. The key features extracted via ML include amplitude, frequency, polarity, and duration. Dimensionality reduction techniques such as principal component analysis (PCA) and linear discriminant analysis (LDA) are commonly employed in data preprocessing. The core objective of ML is to make accurate predictions based on new, unseen data. This necessitates the division of data into training and test datasets, where the training dataset is used for algorithm training and model development, whereas the test dataset assesses algorithm performance. Algorithm selection, hyperparameter tuning, regularization, and optimization are essential preparatory steps before model training commences.^[^ ^181^ ^]^ ML training algorithms traditionally include classification, regression, and clustering, with opportunities for fine‐tuning to enhance performance using parameters such as learning rate adjustment. Upon completing the aforementioned processes, the model becomes capable of making predictions for new datasets. This section of the study delves into a comprehensive exploration of commonly used ML algorithms in wearable systems. Three main categories of algorithms are covered: data preprocessing algorithms (e.g., PCA, LDA), traditional ML algorithms (e.g., Decision Trees (DTs), Support Vector Machines (SVMs), Artificial Neural Networks (ANNs)), and DL algorithms (e.g., Convolutional Neural Networks, Recurrent Neural Networks).^[^ ^181^, ^182^ ^]^

**Figure 10:**
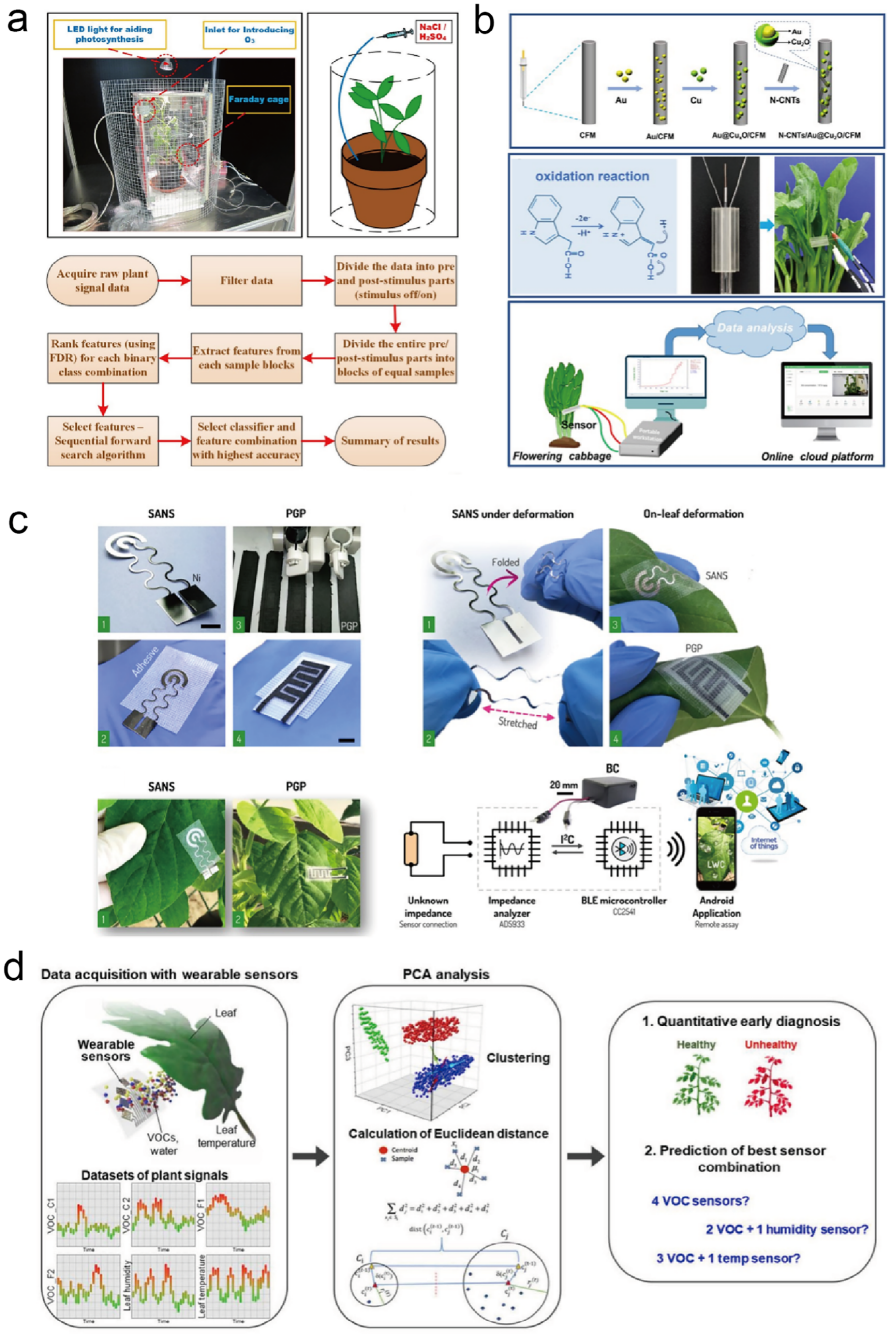
ML‐integrated plant wearable sensors. a) Setup for obtaining plant electrical signals. Reproduced with Permission.^[^ ^186^ ^]^ Copyright 2017, Elsevier BV. b) Schematic of the N‐CNTs/Au@Cu2O/CFM sensors, assisted by ML data analysis. Reproduced with Permission.^[^ ^189^ ^]^ Copyright 2022, American Chemical Society. c) Wearable plant sensors for monitoring water loss in soy leaves. Reproduced with Permission.^[^ ^125^ ^]^ Copyright 2022, American Chemical Society. d) ML approach in an abaxial multimodal wearable plant sensor. Reproduced with Permission.^[^ ^9^ ^]^ Copyright 2023, American Association for the Advancement of Science.

Classification algorithms can be categorized into supervised, semi supervised, and unsupervised. Supervised algorithms, exemplified by SVM and AdaBoost, mandate that all samples used for training the classifier be manually labeled. Unsupervised algorithms, including clustering, operate on unlabeled samples using inherent sample information to perform classification tasks. Semi supervised algorithms, typically derived from supervised algorithms, require that only a portion of the training samples bear class labels. Traditional ML algorithms such as DT, SVM, and ANN tend to exhibit superior performance compared to deep learning models when working with limited data input. This is due to their simpler architectures and lower risk of overfitting in small‐sample scenarios.^[^ ^183^, ^184^ ^]^ DT, with its tree‐like structure, offers solutions to both classification and regression problems.^[^ ^185^ ^]^ The goal of this algorithm is to construct a model that predicts the target variable by deducing data features and learning decision rules. A classification DT model consists of nodes classified as internal and leaf nodes. Internal nodes correspond to features, whereas the leaf nodes represent class labels. To identify the most discriminative features and achieve optimal classification results, each feature in the dataset is evaluated. Subsequently, the original dataset was subdivided into data subsets distributed across all branches, originating from the first decision point. If the data within a branch belongs to the same class, the branch is concluded to be a leaf node, thereby determining the classification. DT is lauded for its simplicity, interpretability, and visualizability, however, it may encounter challenges when processing high‐dimensional data and is susceptible to overfitting as the tree depth increases. DT has found applications in wearable devices, especially in the diagnosis of respiratory ailments. Vitaletti et al. used a DT algorithm for the detection of external chemical stimuli from plant electrical responses (**Figure** **10**a).^[^ ^186^ ^]^ In their study, different plants were exposed to NaCl, O_3_, and H_2_SO_4,_ and their related response signals were collected. Following the application of preprocessing methods, including filtering and drift removal, a limited set of statistical features was obtained from the plant's electrical signals. Using these features along with various classification algorithms, a multiclass classification approach based on DTs was implemented to distinguish among three distinct external chemical stimuli. Their study provided a comprehensive investigation into the creation of a DT classifier using a combination of five distinct discriminant analysis classifiers and 15 statistical features derived from plant electrical signals. Two multiclass classification strategies, one‐versus‐one, and one‐versus‐rest, were applied in their study, and the classifier performance was evaluated through both retrospective and prospective testing. The results revealed that among the three chemical stimuli, NaCl demonstrated the highest degree of separability in most cases as compared to O_3_ and H_2_SO_4_.^[^ ^186^ ^]^ SVMs constitute a class of generalized linear classifiers that perform binary classification in a supervised learning manner.^[^ ^187^ ^]^ Their primary aim is to identify a hyperplane that separates two classes of samples within the input variable space, ideally with a “maximum margin.” By introducing a kernel function, an SVM can handle nonlinear problems adeptly. It excels when processing small‐to‐medium sample datasets, particularly when addressing nonlinear and high‐dimensional classification challenges. However, it has limitations when dealing with extensive training samples. SVMs are frequently employed in wearable sensing systems given that their data typically encompasses small volumes, thus making SVMs an effective choice for performing classification tasks. Prabu et al. used a hybrid SVM to monitor tea leaf disease based on image processing and pattern recognition techniques.^[^ ^188^ ^]^ The images that underwent color transformation were segmented using the watershed algorithm. Subsequently, a multiclass SVM classifier was employed to categorize tea leaf diseases using the gradient feature values extracted from the tea leaf images. ANN is another approach that forms intricately interconnected networks comprising numerous neurons. Each node represents an output function (activation function), and the connections between nodes are weighted for signal propagation. A neural network encompasses an input layer, multiple hidden layers, and an output layer. Neural network operations are divided into forward and backward propagation, involving network structure establishment and parameter training. The design of neural networks revolves around configuring the hidden layers and their interneuron weights. Wu et al.^[^ ^189^ ^]^ introduced an online intelligent monitoring system for indole‐3‐acetic acid (IAA) that comprised a reusable microsensor, a portable electrochemical workstation, and an online cloud platform (Figure 10b). To establish the relationship between electrochemical signals and IAA concentration, an ANN algorithm was used. Subsequently, this ANN model was integrated into the online monitoring system to intelligently determine IAA concentration. The authors mentioned that by using this system, they achieved long‐term real‐time monitoring of IAA concentration in a living cabbage stem over a 12 h period.

Other types of ML approaches were also used to analyze and convert the sensing data collected by wearable plant sensors. In 2022, Barbosa et al. proposed a range of innovative solutions for sensing materials, technology, and data processing to overcome various challenges (Figure 10c).^[^ ^125^ ^]^ The electrodes were prepared from standalone Ni structures (SANSs) and pyrolyzed graphitic paper (PGP) electrodes. These electrodes facilitated the reliable measurement of leaf water content (LWC) in soy leaves with optimized sensitivity, achieving 27.0 kΩ %^−1^ for Ni and 17.5 kΩ %^−1^ for PGP. Additionally, an ML model was employed to translate the sensor responses into a straightforward mathematical equation, accurately assessing water content impairments at two different temperatures (30 and 20 °C) with minimal root‐mean‐square errors (0.1–0.3%). To enhance the accuracy and ensure the quantification of LWC at 20 °C, they employed eight supervised techniques to process the existing Bode plots obtained from long‐term monitoring within the plant growth chamber. Although these techniques yielded satisfactory results, deviations exceeding 25% from the ideal case (100% accuracy) were observed when the single‐frequency analytical curve. Notably, among all the ML approaches applied, multioutput regression and random forest demonstrated the highest levels of accuracy for the data collected at 30 and 20 °C, respectively. Lee et al.^[^ ^9^ ^]^ used a PCA ML model to examine multichannel sensor data, enabling the quantitative identification of the tomato‐spotted wilt virus within as little as four days following inoculation (Figure 10d). The developed model also assessed various sensor combinations for the early detection of disease and suggested that at least three sensors, including VOC sensors, are necessary to achieve early disease detection. Furthermore, the results suggested that, for successful disease detection, the biochemical VOC sensor is likely the most crucial component in each sensor combination. Additionally, the leaf surface humidity sensor exhibited a slightly greater efficacy in disease detection as compared with the leaf temperature sensor. ML analysis can assist in identifying the most influential sensor (and sensor combination) for a specific application, potentially leading to a reduction in the overall number of redundant sensors. This approach could be particularly valuable for lowering sensor costs while preserving sensor performance.^[^ ^9^ ^]^

## 4 Summary and Future Perspectives

**Figure 11:**
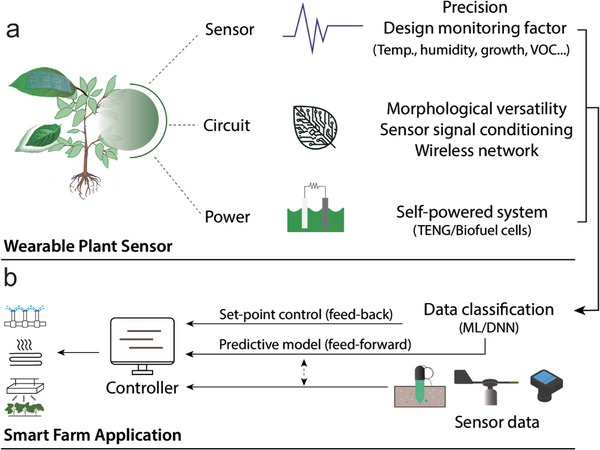
Process of wearable sensor incorporated into smart farming. a) Design elements of a wearable device mentioned in this review – sensor, circuit, and power generator. b) The flow of interaction of wearable sensor data with smart‐farm components and data classification to optimizecontrol elements (irrigation, heating, and lighting) by the control model derived from sensor values. The overall flow follows a solid arrow, and the dotted arrows between the sensor data and the controller represent the interaction for deriving a control model.

We conducted a comprehensive review of the significant developments in wearable plant‐sensing technologies and their use in smart farming. Current agricultural practices use information from multiple sensors to manage abiotic and biotic environments around plants, however, the use of plant sensors can prove to be a significant approach toward achieving more sustainable and efficient resource delivery.^[^ ^190^ ^]^ This review discussed the design and performance of wearable sensors used currently and their applicability to smart farming, focusing on the sensor unit, circuit, power supply, and ML approaches (**Figure** **11**). Detailed insights into the operational principles, designs, and performance characteristics of these sensors were provided and discussed in detail. Consequently, after a comprehensive examination, we anticipate that the primary areas of emphasis in future research on plant sensing will revolve around enhancing the performance of plant sensors, including their sensitivity, specificity, and working range. Sensitivity and specificity play a crucial role in ensuring that sensors can detect subtle changes in plant health or environmental conditions with accuracy. For instance, in soil moisture monitoring, even small variations in water content can indicate plant stress, necessitating sensors with a high sensitivity. Similarly, leaf wetness sensors require the ability to detect changes in humidity to track early‐stage disease indicators.^[^ ^191^ ^]^ If sensitivity is insufficient, these subtle but critical cues may go unnoticed, whereas poor specificity can lead to false positives due to external environmental interference. In addition, the working range of sensors determines their usability across diverse plant species and environmental conditions.^[^ ^59^ ^]^ A limited range can restrict their effectiveness in precision agriculture applications, where variations in temperature, humidity, and soil composition influence plant physiology. Studies have shown that optimizing these factors significantly enhances the accuracy and reliability of plant physiological monitoring, ultimately improving decision‐making in precision agriculture.^[^ ^80^, ^81^ ^]^

Many existing plant monitoring sensors exhibit promising sensitivity levels, but their real‐world performance is affected by external variables such as temperature fluctuations, sensor degradation, and environmental noise, which impact long‐term stability. Similarly, resistive strain sensors can measure leaf deformations within the 0.1% strain range, yet prolonged exposure to environmental stressors can lead to signal drift over time.^[^ ^192^ ^]^ While pH sensors provide reliable readings under controlled conditions, they often require frequent recalibration due to electrode degradation in agricultural field applications. These challenges highlight the need for continuous advancements in sensor materials, calibration techniques, and adaptive data processing methods to enhance long‐term reliability. While strain and pH sensors are indeed well‐established and sufficient for many plant monitoring applications, their integration into wearable sensing systems must also consider additional challenges such as stability, long‐term durability, and multi‐parameter monitoring. Strain sensors can effectively measure plant growth and movement, but their mechanical stability must be maintained over extended periods. Similarly, pH sensors, although widely used for soil and nutrient monitoring, require frequent recalibration, which limits their practicality in autonomous field applications. To improve wearable plant sensors, efforts should focus on enhancing stability, miniaturization, and integration of multiple sensing modalities to enable more comprehensive monitoring. Although current strain and pH sensors provide valuable data, the broader challenges of specificity, environmental adaptability, and real‐time data integration remain key areas for further development.^[^ ^82^, ^86^ ^]^

In this regard, to achieve high strain sensitivity for plant growth monitoring, various approaches, such as wrinkle and crack formation, can be employed to significantly increase sensor sensitivity.^[^ ^100^, ^193^ ^]^ To enhance the strain sensing range, one of the most important approaches includes the introduction of microstructures to the sensor surface.^[^ ^193^ ^]^ Different types of microstructures can be introduced to enhance the sensing operational range and response of plant sensors, including pillars, pyramids, microspheres, porous structures, hollow spheres, and wrinkled formations. To enhance the operational working range of sensors in severe ecosystems, various strategies, including the use of specific organic polymers, have been introduced. Various organic devices, including organic thin‐film transistors and conducting polymer electrodes, have been employed as temperature‐sensing platforms that have broad working capabilities in wearable plant sensors.^[^ ^194^, ^195^, ^196^ ^]^ Wearable devices with higher temperature sensitivity, developed using organic electronic technology, enable precise monitoring of plant physiological responses such as transpiration and stomatal reactions, comparable to traditional sensors used in smart farming. While significant progress has been made in the development of deformation sensors with sufficient sensitivity and dynamic range for plant monitoring, challenges remain in ensuring long‐term stability and minimizing signal drift, particularly under varying environmental conditions. Recent studies highlight that factors such as material degradation, hysteresis, and environmental influences contribute to signal instability, necessitating further research into sensor robustness and compensation mechanisms to enhance reliability in real‐world agricultural applications.^[^ ^197^, ^198^ ^]^

The interface circuit of wearable plant sensors should facilitate in situ monitoring across a wide area. To achieve this, it's imperative to incorporate wireless networks with extended transmission range and low power consumption, as already applied in smart farm technology. However, many studies to date have primarily focused on the operation of the interface circuit to demonstrate their sensors, rather than clarifying power consumption to use the sensor over a long time (Table 1). Therefore, future studies should focus on showing or improving the power consumption of their interface circuits. In addition, enhancing the sensing cycle and data manipulation/storage technology is essential for showcasing precise, long‐term continuous monitoring, a feature predominantly associated with non‐standalone systems, and enabling comprehensive diagnostic results. Periodic sensor calibration is crucial for ensuring ongoing data accuracy and reliability. User interfaces within the interface circuit should offer interaction and data access capabilities. To effectively manage multiple sensors across expansive areas, the use of GPS functionality for precise location tracking is an optimal solution. Ultimately, integrating the circuit into a miniaturized and compact chip is essential for realizing a truly wearable standalone sensing system, minimizing its environmental impact on plants or farming areas. As an initial step in this development, comprehensive discussions regarding the cost, size, weight, retrieving method, and other aspects of the designed interface circuit are encouraged, as executed in a previous study,^[^ ^57^ ^]^ thus enhancing research reproducibility and reliability and accelerating the demonstration of the commercial value of wearable standalone systems. In addition to miniaturizing circuits, research on integrating wearable sensors with interface circuits should also be conducted simultaneously. Wearable sensors are typically thin, flexible, and stretchable, making them sensitive to torsion and the mechanical load of electrodes during connections. In contrast, circuits are often robust but have limited wiring areas. Therefore, a wiring method needs to be developed to seamlessly connect these soft‐hard electronic interfaces without altering their mechanical or electrical signal properties. In the long term, research efforts should also focus on developing biodegradable circuit components, starting with the PCB.^[^ ^199^, ^200^ ^]^ To extract meaningful signals from data collected from plant sensors, ML techniques such as principal component analysis, fuzzy logic, and deep neural networks to solve nonlinearities should be used to increase the robustness of sensors to external factors and reduce uncertainties that may occur in actual smart agriculture. Refined wearable sensor data are naturally embedded in smart agriculture by interacting with off‐the‐shelf smart‐farm components to help implement optimal actuator control loops (Figure 11).

One of the future trends in plant sensors is the fabrication of cost‐effective, reliable, and maintenance‐free sensors that can operate independently or harness self‐sustaining power sources based on working mechanisms, such as TENGs, piezoelectric nanogenerators, and biofuel cells constructed using living organisms. Energy‐harvesting functions can play a crucial role in enabling the widespread adoption of wearable sensors for long‐term field applications. Considering that budget‐friendly fabrication processes can be employed to design organic electronic technologies and that such technologies have demonstrated significant performance in the sensing applications mentioned earlier in this paper, they have emerged as highly practical and viable candidates for the development of cost‐effective wearable sensors designed specifically for monitoring plant microenvironments. Additionally, attributes such as biodegradability, affordability, and multimodality are anticipated to play pivotal roles in advancing plant‐sensing systems for smart farming and precision agriculture technologies in the future.

## Conflict of Interest

The authors declare no conflict of interest.
